# Directional and balancing selection in human beta-defensins

**DOI:** 10.1186/1471-2148-8-113

**Published:** 2008-04-16

**Authors:** Edward J Hollox, John AL Armour

**Affiliations:** 1 Department of Genetics, University of Leicester, Leicester, UK; 2 Institute of Genetics, University of Nottingham, Nottingham, UK

## Abstract

**Background:**

In primates, infection is an important force driving gene evolution, and this is reflected in the importance of infectious disease in human morbidity today. The beta-defensins are key components of the innate immune system, with antimicrobial and cell signalling roles, but also reproductive functions. Here we examine evolution of beta-defensins in catarrhine primates and variation within different human populations.

**Results:**

We show that five beta-defensin genes that do not show copy number variation in humans show evidence of positive selection in catarrhine primates, and identify specific codons that have been under selective pressure. Direct haplotyping of *DEFB127 *in humans suggests long-term balancing selection: there are two highly diverged haplotype clades carrying different variants of a codon that, in primates, is positively selected. For *DEFB132*, we show that extensive diversity, including a four-state amino acid polymorphism (valine, isoleucine, alanine and threonine at position 93), is present in hunter-gatherer populations, both African and non-African, but not found in samples from agricultural populations.

**Conclusion:**

Some, but not all, beta-defensin genes show positive selection in catarrhine primates. There is suggestive evidence of different selective pressures on these genes in humans, but the nature of the selective pressure remains unclear and is likely to differ between populations.

## Background

A rapid response to counter and contain microbial infection is provided by the innate immune system in all multicellular organisms. A key component of the innate immune response is the broad spectrum of antimicrobial proteins that can either be constitutively expressed on epithelia or be induced during inflammation [1,2]. There is evidence for extensive diversity of antimicrobial proteins, with different genes arising by duplication followed by positive adaptive selection [3-5]. Analysing the history of duplication and sequence variation of these antimicrobial genes will help us to understand how selection by infectious diseases has led to functional adaptation at the molecular level.

The beta-defensins are a family of genes which has key roles in the innate immune response, such as antimicrobial activity, antiviral activity and signalling [1]. Some members have also been shown to have an important role in reproduction – for example the protein encoded by *DEFB126 *coats the sperm surface and mediates attachment of the sperm to the oviduct [6,7]. Evolutionary analyses of the beta-defensins have produced evidence for repeated rounds of duplication and divergence of the mature peptide by positive selection. Most convincing are studies which show rapid duplication and divergence of beta-defensin genes since mouse and rat divergence, supporting a birth-and-death model for beta-defensin evolution in the rodents [8,9].

Infectious disease is likely to have been a major selective agent in human evolution [10]. Changes of habitat and of social system during evolution of hominids, and other primates, alter the spectrum of infectious diseases encountered at different times and by different species. Aspects of more recent human evolution are also likely to have been driven by infectious disease: archaeological evidence suggests that individuals in agricultural populations would have suffered more from "crowd diseases" compared to hunter-gatherers, due to the proximity of domestic animals and of each other [11,12]. As well as an increase in infectious disease, the transition from hunter-gatherer to farmer is likely to have had an effect on the genes of the different populations in other ways, such as changes in diet and reproductive behaviour [13].

In order to understand the evolutionary pressures on beta-defensins in primates, we determined putative orthologues of human beta-defensins from six other catarrhine primate species: chimpanzee (*Pan troglodytes*), gorilla (*Gorilla gorilla*), orangutan (*Pongo pygmaeus*), gibbon (*Hylobates lar*), long-tailed macaque (*Macaca fascicularis*) and rhesus macaque (*Macaca mulatta*). We used the known branching order of the primate phylogeny, together with a maximum-likelihood approach using different models of evolution, to test whether positive selection has acted on these genes. Analysing selection in the catarrhine lineage allowed us to identify specific codons in certain beta-defensins that showed a response to environmental change over the last 25 million years. By analysing the variation in these genes in a cohort of hunter-gatherer and agricultural human populations, we infer their more recent evolution and discuss whether population-specific selection processes are responsible for the patterns of variation.

## Results

### Comparative sequence analysis

The beta-defensin gene family is defined by a six-cysteine core motif (C-X_6_-C-X_4_-C-X_9_-C-X_6_-C-C) which was previously used as a motif to search the genome for additional beta-defensins [14]. Some of these were confirmed to encode full-length mRNA transcripts and code for proteins whose amino acid sequence differed considerably outside the shared core motif [14,15]. This coding sequence difference between beta-defensin genes continues into intronic sequence, so that both coding sequence and intronic sequence can define clear orthologues between humans, chimpanzees and rhesus macaques. In addition, according to the current genome assemblies (panTro2 and rheMac2) the expected syntenic relationships are maintained across these three species. By designing primers in non-coding sequences that clearly distinguish between beta-defensin genes, we identified and sequenced putative orthologues in six primate species of coding sequences for all 17 beta-defensin genes for which full-length transcript evidence existed in humans at the start of the project (supplementary figure 1; sequence accession numbers given in supplementary table 1).

**Figure 1:**
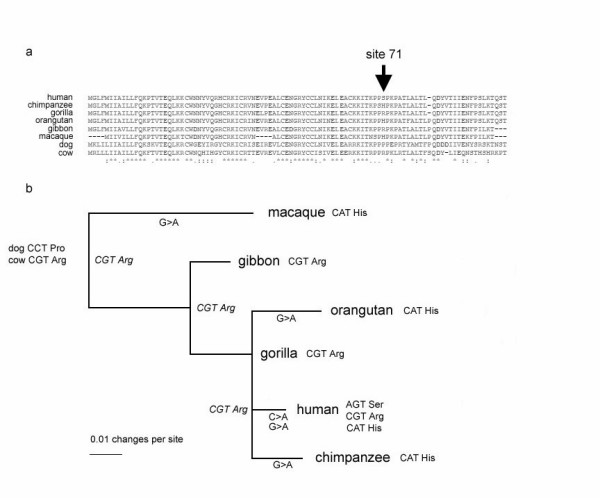
**Analysis of *DEFB127 *evolution**. a) Alignment of predicted amino acid sequences of primate DEFB127 proteins, together with the putative orthologue in dog and cow. Site 71 is highlighted. b) Phylogeny of the *DEFB127 *gene, with branch sites from the species phylogeny (see Methods) and branch lengths generated from the *DEFB127 *nucleotide data by maximum-likelihood. Branches showing codon changes corresponding to amino acid changes at site 71 are highlighted.

Using the information from the known phylogeny of the primate species, we assessed the orthologous sequences for evidence of positive selection. We analysed the ratio (ω) between the non-synonymous substitution rate and the synonymous substitution rate, using a maximum-likelihood technique implemented by the program PAML. This uses models that allow rates of evolution to vary between codons, so allowing the identification of specific positions likely to have undergone positive selection [16,17]. Five genes showed evidence of positive selection: *DEFB1*, *DEFB118*, *DEFB120*, *DEFB127 *and *DEFB132 *(table 1). Codon 71 of *DEFB127 *has been positively selected and is very variable between species (figure 1a), and analysis of ancestral sequences suggests that this codon has independently mutated five times during primate evolution (figure 1b).

**Table 1:** Maximum-likelihood codon substitution analyses of beta-defensin genes in catarrhine primates

Location	Gene	Number of aligned codons	Likelihood Model M1a 0<ω_0_<1, ω_1 _= 1	Likelihood Model M2a 0<ω_0_<1, ω_1 _= 1, ω_2_>1	Positive selection P value	Selected sites (probability)
8p23.1	*DEFB1*	67	-397.25	-390.07	0.00076*	R61 (0.997)
	*DEFB4*	63	-401.51	-400.69	0.44	
	*DEFB103*	66	-334.85	-334.85	1.0	
	*DEFB104*	71	-407.87	-406.55	0.27	
	*DEFB105*	77	-509.99	-509.99	1.0	
	*DEFB106*	64	-353.50	-353.09	0.66	
	*DEFB107*	65	-343.19	-343.19	1.0	
20q11	*DEFB118*	122	-734.34	-728.92	0.0044*	R110 (0.967)
	*DEFB119*	83	-501.92	-501.23	0.50	
	*DEFB120*	86	-485.55	-478.39	0.00077*	M81 (0.998)
	*DEFB123*	66	-289.31	-289.25	0.95	
20p13	*DEFB125*	125	-779.12	-779.10	0.98	
	*DEFB126*	85	-591.65	-591.47	0.83	
	*DEFB127*	87	-498.13	-493.43	0.0091*	S71 (0.984)
	*DEFB128*	91	-526.11	-526.09	0.98	
	*DEFB129*	181	-946.41	-946.41	1.0	
	*DEFB132*	93	-594.10	-590.54	0.029*	I57 (0.950)

### Copy number analysis

We and others have previously shown a cluster of beta-defensin genes (*DEFB4*, *DEFB103*, *DEFB104*, *DEFB105*, *DEFB106*, *DEFB107*) to be copy number variable in humans; the neighbouring *DEFB1 *gene is not involved in this copy number variation [18-20]. It is essential to understand whether a gene is copy number variable in order to correctly interpret apparently heterozygous SNP sites, and to be aware of the possibility of gene conversion between closely related paralogues.

We used multiplex amplifiable probe hybridisation (MAPH) to examine whether other members of the beta-defensin family varied in copy number, in addition to those previously described. MAPH is a method for gene copy number analysis that relies on quantitative recovery of amplifiable probes following hybridisation to genomic DNA [21,22]. Using MAPH probes for the beta-defensin gene regions analysed in this study, we asked whether there was any evidence of copy number variation in the 37 DNA samples used for the population analyses described in this paper, and in a further 153 individuals from the UK. We reasoned that variation between duplicate tests of individuals would be highly correlated where underlying copy number variation was the main cause of the variation, but less correlated, if at all, when the main cause was random experimental noise. The correlation between the two sets of raw MAPH results for 190 samples was calculated, and the probes mapping to the copy number variable region at 8p23.1 showed high correlation coefficients (0.70<r<0.96). All other beta-defensin probes showed correlation coefficients less than 0.52 (supplementary table 2), and there was no grouping of samples on scatterplots indicative of rarer deletion or duplication polymorphisms.

### Population analyses

The second exon, which codes for the entire mature peptide, was resequenced from all five beta-defensins that showed positive selection, in a panel of 37 individuals from four human populations. Twenty polymorphic sites were found, of which 16 are predicted to change an amino acid, and only 5 are present in dbSNP release 126 (rs16995685, rs12624954, rs2738047, rs16995668, rs1800957 - please see NCBI webpage in Availability and requirements section).

The number of synonymous and non-synonymous polymorphic sites and fixed differences between the human and macaque exon 2 sequence was determined (table 2). A McDonald-Kreitman test was performed on each gene, and on the pooled results, to test for an excess of non-synonymous fixed substitutions which suggests the influence of positive selection of the gene [23]. None of the tests showed significant evidence of selection (data not shown), in contrast to the results obtained from the ω-tests using PAML. This difference could be due to the small number of changes and polymorphic sites in each test, which is a reflection of the small size of the genes; but pooling the data from all five genes also failed to show a significant result. More plausibly, the discrepancy could be due to the high numbers of non-synonymous polymorphisms identified in humans (16 in total, compared with 4 synonymous polymorphisms). Five of these are singletons, but eleven were identified in two or more samples, suggesting that they may be at appreciable frequencies in different populations.

**Table 2:** Human-macaque divergence and human coding polymorphisms of genes positively selected in catarrhine primates.

Gene	Human – macaque non-synonymous fixed changes	Human – macaque synonymous fixed changes	Human non-synonymous polymorphisms	Human synonymous polymorphisms	Rs number	Human non-synonymous polymorphic sites	Indigenous Australians	UK population	Other sub-saharan Africans	Mbuti/Biaka	Agriculturalists	Hunter-Gatherers	Fisher's exact test p value (not corrected for multiple tests)	African	Non-African	Fisher's exact test p value (not corrected for multiple tests)
DEFB127	8	6	4	1	16995685	R71S	4	8	0	1	8	5	0.175	1	12	**0.002**
					-	R71H	0	0	0	2	0	2	0.288	2	0	0.208
					12624954	G31R	4	8	0	1	8	5	0.175	1	12	**0.002**
					-	K58R	0	0	1	0	1	0	0.459	1	0	0.459
DEFB120	13	2	3	0	-	H28R	0	0	1	3	1	3	0.371	4	0	**0.040**
					-	R46C	1	0	0	0	0	1	0.541	0	1	0.541
					-	*89EL*	0	0	1	3	1	3	0.371	4	0	**0.040**
DEFB132	12	8	5	2	-	W41S	0	0	1	2	1	2	0.561	3	0	0.092
					-	P63Q	0	0	3	1	3	1	0.248	4	0	**0.040**
					-	L70F	0	0	1	2	1	2	0.561	0	3	0.152
					-	V93I/T	4	0	0	2	0	6	**0.021**	2	4	0.418
					-	V93A/T	3	0	1	9	1	12	**0.002**	10	3	**0.015**
DEFB118	11	7	2	0	-	C34R	0	1	0	0	1	0	0.459	0	1	0.541
					-	I56V	0	0	1	4	1	4	0.234	5	0	**0.017**
DEFB1	6	7	2	1	-	F24C	0	0	0	1	0	1	0.541	1	0	0.459
					2738047	V38I	0	0	0	1	0	1	0.541	1	0	0.459
n	50	30	16	4	-	16	20	20	14	20	34	40	-	34	40	-

We analysed the frequencies of the polymorphisms in four different population samples: hunter-gatherers Mbuti/Biaka (MB) and Indigenous Australian (IA), and agriculturalists British (UK) and sub-Saharan African (SSA) (table 2). A Fisher's exact test of independence between Africans and non-Africans reveals significant differences in allele frequency for many polymorphisms, as expected. The strongest differences in allele frequencies were for the R71S and G31R polymorphic sites in *DEFB127*. Two DNA polymorphisms, both affecting codon site 93 in *DEFB132*, showed significantly different frequencies between agricultural and hunter-gather populations.

### Analysis of *DEFB127 *and *DEFB132*

We decided to study the haplotype structure of both *DEFB127 *and *DEFB132 *genes by complete resequencing. For *DEFB132*, resequencing 292 bases spanning exon 2 from 74 chromosomes revealed 10 polymorphic sites associating to form 14 haplotypes, inferred from diplotype data by maximum-likelihood analysis (supplementary table 3). For *DEFB127*, resequencing 3208 bases spanning exons 1 and 2 in 74 human chromosomes revealed 36 polymorphic sites associated to form 30 haplotypes, determined unambiguously by allele-specific PCR (supplementary table 4). We analysed the forces affecting variation at different loci by calculating various summary statistics from the haplotype data at both *DEFB127 *and *DEFB132 *in each population (table 3). Both genes show high values for gene diversity (π), indicating a high degree of heterozygosity in all populations. There are four estimates of the population mutation parameter (θ), derived from levels of homozygosity (θ_H_), number of segregating sites (θ_s_), nucleotide diversity (θ_π_), and the expected number of alleles (θ_k_).

**Table 3:** Within-population diversity metrics for *DEFB127 *and *DEFB132*. p values are shown when less than 0.05, error values represent standard deviations unless otherwise indicated

Gene	Metric	Indigenous Australians	UK	Other sub-Saharan Africans	Mbuti/Biaka
DEFB127	nucleotides	3210	3210	3210	3210
	Gene Diversity	0.72 +/- 0.089	0.83 +/- 0.057	0.97 +/- 0.036	0.95 +/- 0.033
	Segregating sites	22	22	13	30
	Nucleotide diversity (π) ×10^-3^	2.3 +/- 1.3	3.2 +/- 1.7	1.4 +/- 0.8	1.5 +/- 0.8
	θ_H_	2.0 +/- 0.93	4.0 +/- 1.8	28 +/- 33	18 +/- 14
	θ_s_	6.2 +/- 2.4	6.5 +/- 2.5	4.1 +/- 1.8	8.4 +/- 3.2
	θ_π_	7.5 +/- 4.1	10 +/- 5.5	4.5 +/- 2.6	4.7 +/- 2.7
	θ_k _(95% confidence limits)	2.5 (0.97 – 6.2)	4.4 (1.8 – 10)	22 (8 – 66)	19 (8.2 – 47)
	Fu's F_s_	4.63	3.63	-4.35	**-5.38 (p = 0.013)**
	Tajima's D	0.82	**2.27 (p = 0.030)**	0.38	**-1.72 (p = 0.030)**
	Fay and Wu's H	-2.31	1.05	-0.13	**-9.61 (p = 0.019)**
	Nucleotide divergence human-chimp %	1.2+/-0.2			
DEFB132	nucleotides	292	292	292	292
	Gene Diversity	0.69 +/- 0.11	0.56 +/- 0.063	0.73 +/- 0.10	0.87 +/- 0.048
	Segregating sites	5	7	7	10
	Nucleotide diversity (π) ×10^-3^	6.9 +/- 4.5	5.5 +/- 3.8	7.2 +/- 4.8	10.3 +/- 6.3
	θ_H_	0.97 +/- 0.25	1.7 +/- 0.87	2.0 +/- 1.1	5.8 +/- 2.7
	θ_s_	1.1 +/- 0.65	1.4 +/- 0.76	2.5 +/- 1.2	2.5 +/- 1.2
	θ_π_	1.6 +/- 1.1	2.0 +/- 1.3	2.1 +/- 1.4	3.0 +/- 1.8
	θ_k _(95% confidence limits)	0.72 (0.21 – 2.3)	3.4 (1.4 – 8.1)	2.3 (0.81 – 6.4)	4.4 (1.8 – 10)
	Tajima's D	1.29	1.25	-0.62	0.65
	Fu's F_s_	-1.20	2.51	0.19	-0.83
	Fay and Wu's H	-0.095	0.35	-2.42	-0.60
	Nucleotide divergence human-chimp %	2.8+/-1.0			
Number of chromosomes		20	20	14	20

Tajima's D compares the distribution of rare frequency alleles with intermediate frequency alleles by comparing θ_s _with θ_π_. *DEFB127 *gives a significantly negative value for the MB and a significantly positive value for the UK population, which may indicate balancing selection. However, this statistic is known to be sensitive to demographic changes: a negative value may result from a population expansion and a positive value from population subdivision. Fay and Wu's H statistic compares the distribution of common frequency alleles with intermediate frequency alleles by comparing θ_H _with θ_π_, and is more robust to demographic changes [24]. For *DEFB127*, D, H, and Fu's F_s _are all significantly negative for the MB population, which suggests directional selection in this population. For *DEFB127*, Tajima's D is significantly positive in the UK population reflection a signal of either balancing selection or population subdivision.

### Mismatch analysis

**Figure 2:**
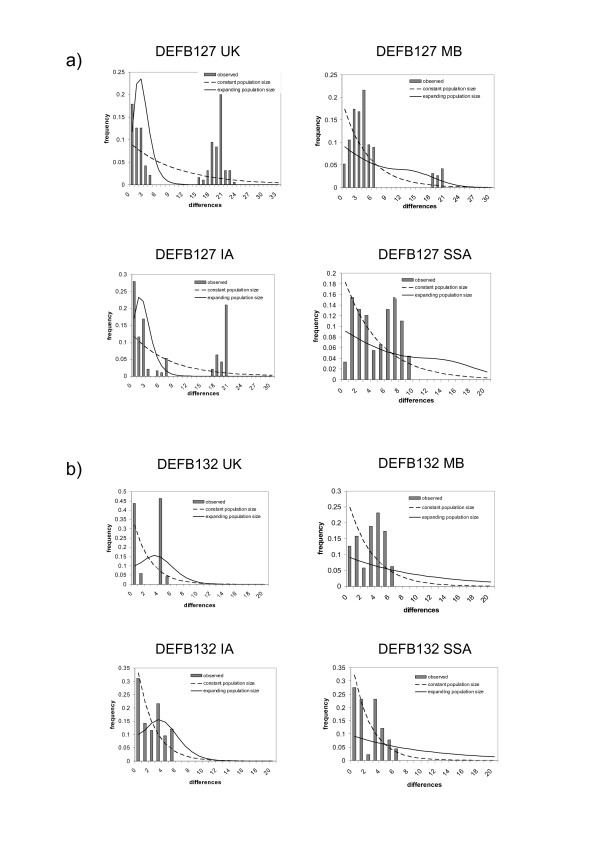
**Mismatch analysis of *DEFB127 *and *DEFB132 *haplotypes**. Mismatch analysis plots are shown for each population, together with a simulated plot assuming neutrality and a sudden population expansion. 95% Confidence intervals to the simulated values were determined by bootstrap analysis. *a) DEFB127. b)DEFB132*.

A mismatch distribution is a histogram showing the number of mismatches when all haplotypes are compared with each other, and describes the haplotype structure of a region. Figure 2a shows the mismatch distributions for *DEFB127 *and figure 2b shows the mismatch distributions for *DEFB132*. On each graph the expected distribution given neutrality with a constant population size is shown (dashed line), and the expected distribution given a recent population expansion is also shown (solid line).

For *DEFB127*, all distributions are characterised by a strong bimodal pattern inconsistent with a model reflecting neutrality, constant population size or a population expansion. In each distribution, the second peak is at around 20 differences, with the exception of SSA, where the second peak is at around 8 differences. The strongest bimodal distribution is in the UK population, reflecting two deep haplotype clades at similar frequency, and suggests balancing selection at this locus in this population. This supports the result from the Tajima's D test. For *DEFB132*, the SSA, UK and MB populations show a bimodal distribution also inconsistent with a model reflecting neutrality, constant population size or a population expansion. For the IA population, the distribution is more consistent with neutrality, either with a constant population size or an expansion.

### Network analysis

**Figure 3:**
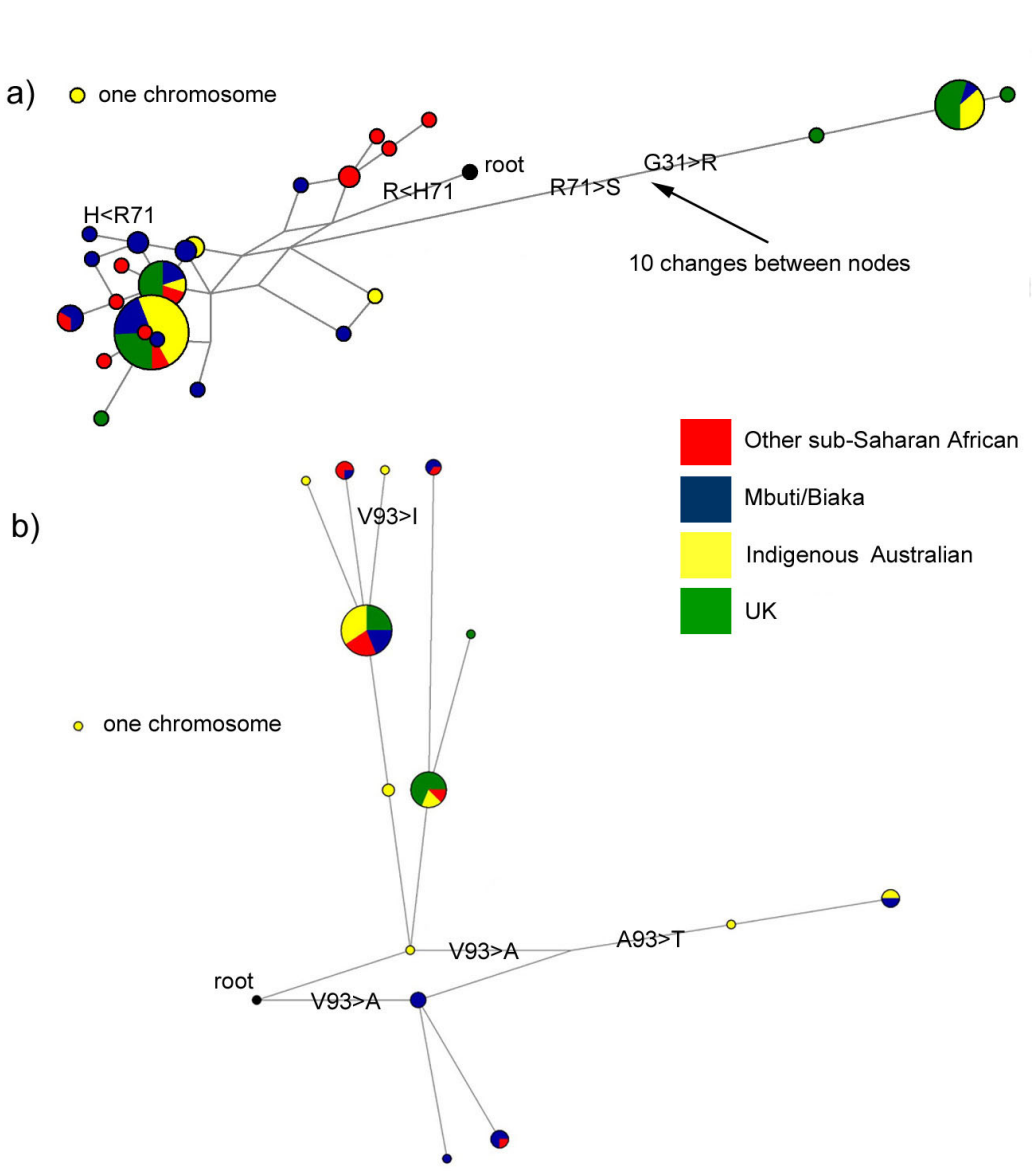
**Network diagram of haplotypes**. Pie size is proportional to number of observations of each haplotype. The number on each branch reflects the polymorphic site, as indicated in supplementary tables 3 and 4. Branches that change an amino acid are also highlighted with that change. a) *DEFB127. b) DEFB132*.

Network diagrams can be used to show haplotype structure graphically. Each haplotype is a node on the diagram linked to other haplotypes that differ by one nucleotide position. They also convey haplotype frequency and distribution information by the size and composition of the pie chart at each node. Analysis of the haplotype network for *DEFB127 *reveals that the bimodal mismatch distribution reflects haplotypes carrying a glycine at position 31 and an arginine at position 71 (G31R71) and a highly diverged haplotype clade carrying an arginine at position 31 and a serine at position 71 (R31S71) (figure 3a).

The *DEFB132 *haplotypes are shown on a network diagram together with the root haplotype (figure 3b). There are four coding alleles at codon 93: valine, which is ancestral and the most common; alanine, which is observed in MB population and one SSA individual; threonine, seen only in the IA and MB; and isoleucine, seen in one IA individual. This suggests that some ancient haplotypes carrying the coding variation at codon 93 have persisted in geographically dispersed hunter-gatherer populations. This could be due to absence of purifying selection that has operated in agricultural populations [25]. The network shows a reticulation and several branches caused either by recurrent mutation or recombination – only polymorphisms 4, 5, 6 and 7 occur on one branch of the network.

### Genomic region analysis

Analysis using HapMap data [26] shows that both diverged *DEFB127 *haplotypes are present in European, Chinese and Japanese, extending as an essentially non-recombining haplotype block for about 25 kb, but neither the R31S71 haplotype nor its constituent alleles are found in the Yoruba from Nigeria (data not shown). Using the CEPH HapMap data, an EHH test was performed to analyse for signatures of recent selection [27]. No haplotype has extended haplotype homozygosity with respect to the other, suggesting that the observed frequencies were not generated by recent positive selection for one or other haplotype (data not shown). It is unclear why the R31S71 haplotype is not seen in the SSA population nor in the Nigerian Yoruba HapMap population. There are three possible explanations: that it has been carried across the world in the out of Africa expansion, and has been lost in certain populations by drift; that it has been maintained in certain populations by balancing selection; or it is a product of a relatively recent hybridisation event.

To exclude selection at another linked gene being responsible for this haplotype pattern, we first examined the extent of linkage disequilibrium across this region in the CEPH HapMap samples. The only other gene on the same haplotype block, and therefore possibly associated with *DEFB127*, was *DEFB126*, 15 kb away. Resequencing of exon 2 of *DEFB126 *found two deletion polymorphisms which substantially altered the C-terminal tail amino acid sequence of *DEFB126 *(Hollox and Armour, unpublished data). These were candidate functional polymorphisms that could have caused a selective sweep affecting polymorphism frequencies and association at *DEFB127*. To investigate this, we genotyped 141 Northern European individuals for the two polymorphisms at *DEFB127 *(G31R and R71S) and the two deletion polymorphisms in *DEFB126*. Maximum-likelihood estimation found no evidence of linkage disequilibrium between the markers at *DEFB127 *and at *DEFB126 *(r^2 ^= 0.05), although there was very strong association (r^2 ^= 0.98) between the two polymorphic sites within *DEFB127 *(data not shown). Therefore there is no evidence of a selective sweep driven by these variant alleles at *DEFB126*.

## Discussion

We have identified five beta-defensin genes that have undergone positive selection in primates, including *DEFB118 *and *DEFB1*, whose protein products show evidence of direct antimicrobial action in urogenital tissues [28,29]. The selected position in *DEFB1 *has been shown to be directly involved in antimicrobial activity and signalling [30], and natural selection has modulated its charge and polarity in response to the microbial environment. The results broadly agree with a previous analysis of the 8p23.1 cluster [31]: we agree that there is no support for the previously suggested effect of selection on the amino acid sequence of *DEFB4 *[32]. More generally, we note that the genes on 8p23.1 that are variable in copy number among humans show no evidence of positive selection in the last 25 million years. This could be because the copy number variable beta-defensins tend to be shorter, and the analyses lack power when analysing short coding sequences. Alternatively, there could be a causal association between copy number variation and the likelihood of coding sequence diversification by positive selection. In primates and the dog, the chromosome 8 cluster does not seem to have undergone as much duplication and rapid divergence since species divergence as rodents [31,33]. In horses, there appears to have been a rapid duplication and divergence of the *DEFB4 *orthologue [34], and a similar process may have occurred in goats [35]. It also appears that the *DEFB106 *and *DEFB107 *orthologues have been positively selected in the Old World Monkey *Cercopithecus aethiops *[31], so further investigation in different catarrhine clades, as well as platyrrhines and other primates, is necessary. The question remains whether, in copy number variable genes, extensive divergence is prevented by purifying selection and gene conversion, or by balancing selection maintaining a pool of similar variants that are shuffled by gene conversion. Determining whether the same loci are copy number variable loci between different primates, and whether copy number variation exists in other mammals, will help to answer this question.

We have used a top-down evolutionary approach to identify variation in genes that is likely to be functional, and has been driven by selection. In doing so, we have revealed high levels of beta-defensin diversity in human populations, but the forces driving this diversity are difficult to identify. *DEFB127 *diversity involves two positions that distinguish two deep-rooting haplotypes, suggesting that there are epistatic interactions between the two amino acids and that both haplotypes have been preserved by balancing selection. Balancing selection has been suggested to maintain variation at several other innate immune genetic loci, such as CD209L cell-surface receptor [36] and the killer immunoglobulin-like receptors (KIR) [37], and at loci involved in reproduction, such as zonadhesin [38]. By chemical synthesis, reconstruction and folding of the core N-terminal sequence, DEFB127 was shown not to have antimicrobial activity [39]. However, *DEFB127*, like several other beta-defensins, is expressed in the epidydimis and may be involved in sperm maturation or reproduction. Evidence for directional selection in the Mbuti/Biaka population indicates contrasting selection pressures in different populations due the different environmental pressures that these populations have faced. Whether balancing selection has been a major force in the human genome is disputed, and distinguishing signatures of balancing selection from neutral variation is difficult without a clear prior hypothesis concerning the potential mechanism of balancing selection in a given candidate gene [40].

*DEFB132 *has been positively selected in the catarrhine lineage, and is highly variable in human populations. However, in humans there is only evidence from mismatch distribution analysis of any statistical departure from neutral evolution at this locus, though the power of the analyses could be limited by the small region analysed. One codon is polymorphic in hunter gatherer populations, coding for four amino acids (V/A/T/I), but in agricultural populations 39 out of 40 chromosomes carry a valine at this position. Genetic differentiation tests reveal a similar contrast between agricultural and hunter-gatherer populations; as expected African and non-African populations are significantly differentiated for both *DEFB127 *(H_st _permutation test, p = 0.008) and *DEFB132 *(p < 0.0001), but for *DEFB132 *the agricultural and non-agricultural populations are also differentiated (p < 0.0001). The basis for any selective pressure is unclear:*DEFB132 *is expressed in the testis, and possibly elsewhere, is secreted and is likely to be antimicrobial although that has not been directly tested [15].

We have shown that beta-defensins have different patterns of selection, and one of the selected amino acids has been shown to be antimicrobial and involved in cell-cell signalling. The challenge will be to relate evolutionary changes in amino acid sequences to differences in antimicrobial, cell-signalling or sperm function. Both a comprehensive picture of the evolution of these genes by identifying orthologues in other mammalian species, and a concurrent effort into characterising the function of these orthologues, is needed in order to fully understand the role of this gene family in innate immunity and reproduction.

## Conclusion

Several beta-defensin genes show positive selection in catarrhine primates, including *DEFB127*, *DEFB118 *and *DEFB1*, but not the beta-defensin genes involved in common copy number variation on chromosome 8p23.1. There is suggestive evidence of different selective pressures on these genes in humans, with *DEFB127 *showing evidence of balancing selection. Given the multifunctional role of the beta-defensin family, however, the nature of the selective pressure remains unclear and is likely to differ between populations.

## Methods

### DNA samples

Genomic DNA from five primates (*Macaca fascicularis *– long-tailed macaque, *Hylobates lar *– gibbon, *Pongo pygmaeus *– orangutan, *Gorilla gorilla *– lowland gorilla, *Pan troglodytes *– common chimpanzee) was extracted from lymphoblastoid cell lines at the European Collection of Animal Cell Cultures (ECACC, Salisbury, UK). DNA from 10 native British individuals (UK) was chosen randomly from the ECACC control panel 1, DNA from 10 Indigenous Australian individuals (IA) and 7 sub-Saharan Africans (SSA, 5 Zulu, 1 Kikuyu, 1 Ghanaian) was obtained from ECACC, and DNA from 10 Mbuti/Biaka "Pygmy" individuals living in the Democratic Republic of Congo (MB) was obtained from Coriell Cell Repositories (New Jersey, USA). DNA from 153 British individuals resident in non-urban Nottinghamshire was used for copy number analysis, and was from a cohort described previously [41].

### PCR, sequencing, and copy number analysis

20 ng of genomic DNA was amplified using a standard PCR mix: 0.2 mM each of dATP, dCTP, dGTP, dTTP, 1 mM MgCl_2_, 75 mM Tris-HCl (pH 8.8 at 25°C), 20 mM (NH_4_)_2_SO_4_, 0.01% (v/v) Tween 20 in a final volume of 20 μl, with 10 pmol of each primer, 0.5 units Taq DNA polymerase (ABgene), followed by thermal cycling for 35 cycles at 95°C 30 seconds, 58°C 1 minute 70°C 1 minute. Amplification from human DNA successfully produced a single band and lower annealing temperature (typically 5°C less than the calculated T_m _at 50 mM salt) for the first 5 cycles was used to minimise the risk of unexpected allele- or paralogue-specific amplification in non-human samples. If, after repeated reduction in annealing temperature, amplification did not result in a single band, primers were redesigned. In a few cases, amplification products could not be generated.

PCR products were prepared for sequencing either using a Gel Extraction kit (Qiagen), eluted in water, or the Ampure SPRI method (Agencourt Bioscience). 5 μl of the solution (~100–250 ng) was then sequenced using BigDye 3.1, following the manufacturer's instructions (Applied Biosystems). Free dye was removed either by ethanol precipitation, or by the Cleanseq SPRI method (Agencourt Bioscience). Sequencing reactions were electrophoresed on an ABI 3730 capillary sequencer by the DNA Sequencing Service at the Biochemistry Department, University of Oxford, and analysed manually using FinchTV (Geospiza). Putative orthologue coding sequences were assembled by alignment with human sequences and submitted to EMBL sequence database (accession numbers in supplementary table 1). In all cases, human DNA was amplified and sequenced using the same primers as for the other primates, and confirmed to be from the correct gene using BLAT against the human genome assembly build 34. *M. mulatta *orthologues were identified from the genome assembly by BLAT searching using the *M. fascicularis *DNA sequence. In all cases, one unique match was identified.

Population resequencing was carried out essentially as above. All polymorphic sites which were only seen as heterozygotes or singletons were sequenced off both strands in those individuals.

Multiplex Amplifiable Probe Hybridisation (MAPH) and Restriction Enzyme Digest Variant Ratios were performed as previously described [22,42], with fluorescent detection of amplification products by an Applied Biosystems 3100 Genetic Analyzer. MAPH involves hybridisation of short amplifiable probes to genomic DNA immobilised on a nylon filter. Following stringent washing, the bound probes are released and amplified using a pair of primer sites common to all probes. One primer is fluorescently labelled so that the resulting PCR product can be run on a capillary electrophoresis machine and quantified. The area under the probe peak is directly proportional to the number of sites for that probe in the immobilised genomic DNA.

We constructed a set of MAPH probes consisting of test probes mapping to individual beta-defensin genes and reference probes that have been shown to be non-variable in copy number [43]. The values for each test probe were normalised for lane intensity against the reference probes, and duplicate values plotted on a scatterplot and the Pearson correlation coefficient (r) calculated for each probe across all tested samples. Scatterplots were also visually inspected for outliers along the x = y diagonal axis. The error rate was calculated by extrapolating the mean (μ = 1) and standard deviation (σ = 0.08) of the duplicate samples of the reference probes to a predicted distribution of measurements of duplication alleles with mean 1.5 μ and standard deviation σ √ 1.5. Assuming a normal Gaussian distribution, the probability of obtaining a value less than 1.3 (threshold for detecting duplications) from this distribution is 2%.

The MAPH probe for *DEFB127 *did not give reproducible results, and copy number variation at this gene was excluded by performing REDVR on both the G31R (rs12624954) and R71S (rs16995685) polymorphic sites. REDVR involves PCR amplification across a restriction enzyme site that varies between copies with a pair of primers, one being fluorescently labelled. This is followed by restriction enzyme digestion and quantification of the two peaks produces a ratio of cut to uncut products.

### Haplotype determination

In order to determine haplotypes at *DEFB127 *from individuals who were heterozygous at more than one site, allele specific PCR (AS-PCR) was performed using primers with a 3'LNA base (Sigma-Proligo), which enhances pyrimidine-purine mismatch determination [44]. Polymorphic sites used for AS-PCR are shown in supplementary table 4. PCR products from both haplotypes were sequenced.

For *DEFB132*, haplotypes were inferred from the diplotype data using the maximum-likelihood Bayesian approach implemented by PHASE2 [45]. 13 samples were heterozygous at two or more sites, and the phase probabilities of all inferred sites was >0.95, with the exception of four sites where the probabilities were 0.93 and 0.94. For comparison, inferring haplotypes at *DEFB127 *from the diplotype data resulted in 19 inferred sites with phase probabilities less than 0.90. This is likely to be due to the high number of haplotypes in Africans that were observed in only one individual, and that differ from each other by more than one site (supplementary figure 4a).

### Interspecific analyses

Putative orthologous DNA sequence of each beta-defensin was aligned by ClustalW using the default parameters except a low gap extension penalty (0.05), using the computer server at the European Bioinformatics Institute (see Availability and requirements section for URL). Phylogenetic trees were constructed from alignments by maximum-likelihood using the program DnaML, part of the Phylip 3.6 suite of programs [46]. Following assembly of the coding sequences of each gene, the protein sequence was aligned using ClustalW with default parameters. This protein alignment was used to inform the codon-based alignment used by the maximum likelihood codon-based selection program PAML 3.14 [16]. Start and stop codons, and deletions, were removed from the codon alignment.

An unrooted tree representing an accepted phylogeny of the primates [47] was used in PAML – branch lengths were not specified, and it was assumed that there was no difference in selective pressures across the branches (one ω ratio). The proximity of nodes for human, chimpanzee and gorilla raises the possibility that the phylogeny for each gene is not the same as the phylogeny of the species, where gorillas diverged first. In order to clarify the phylogeny at *DEFB127*, we resequenced the 3.2 kb spanning the gene in chimpanzee and gorilla. There were no phylogenetically informative sites that allowed us to unambiguously determine the human-chimpanzee-gorilla branching pattern. Therefore the tree was described initially as a trifurcation in input files and the branching pattern allowed to vary as a free parameter so that results were not dependant on constraining the tree to a specific branch pattern at this node.

By fitting the data to two likelihood models in PAML, M1a and M2a, we have a test of whether positive selection, as defined by ω >1, has acted on a gene. M1a is the model assuming a null hypothesis that all codons fall into two classes: either evolving neutrally (ω = 1) or with mild purifying selection (0< ω <1). M2a allows codons to fall into another class: those showing positive selection (ω >1). Therefore, by comparing the log likelihood of the data fitting model M1a with the log likelihood of the data fitting model M2a, a significance level is obtained testing the hypothesis that certain codons are under positive selection. The significance value is derived by calculating 2 × (log likelihood M1a – log likelihood M2a), and comparing the figure with a χ^2 ^distribution with 2 degrees of freedom. P < 0.05 is regarded as significant, with a Holm correction for multiple tests [48].

Identification of codons that have experienced selection were identified by a Bayes Empirical Bayes method implemented in PAML [49]. Briefly, the method calculates the posterior probability that a given codon falls into the ω >1 class, as identified by the M2a model, accounting for the maximum-likelihood estimate sampling errors of the parameters in the model. Ancestral sequence reconstruction at each node, with probabilities at each codon site, is also provided as output by PAML. Branch lengths on the tree in figure 1 were generated by maximum likelihood analysis of the coding sequence by baseml, part of PAML, and the *DEFB127 *primate ancestral codon state was determined by a PAML codeml analysis of the primate sequence, together with *Canis familiaris *and *Bos taurus *orthologues.

### Population genetic analyses

All estimates were performed using Arlequin 3.0 [50] or DnaSP 4.0 [51], with haplotype reduced-median network diagrams drawn using Network 4 (see Availability and requirements section for URL)[52]. Significance for the Tajima's D [53], Fu's F_s _[54] and Fay and Wu's H [24] were generated by comparing the observed distribution against a simulated distribution generated by 1000 runs of a coalescent simulation algorithm, assuming population equilibrium and selective neutrality, with θ taking the value of θ_s _which is calculated from the observed data (table 3).

Genetic differentiation was analysed initially by 2 × 2 contingency tables of individual allele frequencies between population groups, with significance assessed by Fisher's Exact Test. A haplotype-based test, H_ST_, was also used, with significance assessed using a permutation test implemented by DnaSP 4.0 [55].

Mismatch distributions were calculated using DnaSP 4.0. The expected distribution for an expanding population was generated using the following population parameters justified by published studies on human autosomal neutral loci [56]: pre-expansion population mutation parameter (θ_0_) = 1, post-expansion population mutation parameter (θ_1_) = 10. The time from expansion (τ) was estimated using the equation τ = 2 ut, where u is the haplotype mutation parameter and t is time in generations. u was estimated from the human-chimpanzee divergence at each locus, K, given a divergence time of 6 million years [57]. In African populations the expansion time was 1 Mya reflecting the expansion after the speciation of modern humans, and in non-African populations the time was 100 kya, reflecting the expansion out of Africa, and a generation time of 20 years was assumed.

## Availability and requirements

NCBI: http://www.ncbi.nlm.nih.gov/projects/SNP/

European Bioinformatics Institute: http://www.ebi.ac.uk/tools/clustalw

Network 4: http://www.fluxus-engineering.com

## Authors' contributions

EJH participated in the conception and design of the study, carried out all experiments and performed all analyses, and drafted the manuscript. JALA participated in the conception and design of the study, in its coordination, and in the writing of the manuscript.

## Supplementary Material

Additional file 1 **Supplementary Figure 1 Protein alignments of primate beta-defensins**. ClustalW alignments of protein sequences predicted from the DNA sequence are shown. Selected sites, as identified by PAML, are indicated "S", and for sites polymorphic in humans in *DEFB1*, *DEFB118*, *DEFB120*, *DEFB127*, *DEFB132*, are indicated "P". Click here for file

Additional file 2 **Supplementary Table 1 Accession numbers of primate defensin sequences**. Refererence cited in the table is [32]. Click here for file

Additional file 3 **Supplementary Table 2 MAPH probes designed for copy number analysis**. References cited in the table are [43,18,19,58]. Click here for file

Additional file 4 Supplementary Table 3 *DEFB132 *haplotypes. Click here for file

Additional file 5 Supplementary Table 4 *DEFB127 *haplotypes. Click here for file
