# Supplementary material for: Directional and balancing selection in human beta-defensins
Source: BMC Evol Biol. 2008 Apr 16;8:113. doi: 10.1186/1471-2148-8-113 (PMC2373304; doi:10.1186/1471-2148-8-113)
Supplement: Additional file 1 — Supplementary Figure 1 Protein alignments of primate beta-defensins. ClustalW alignments of protein sequences predicted from the DNA sequence are shown. Selected sites, as identified by PAML, are indicated "S", and for sites polymorphic in humans in DEFB1, DEFB118, DEFB120, DEFB127, DEFB132, are indicated "P". [file 1471-2148-8-113-S1.pdf]

Supplementary figure 1

```
DEFB1_hsa        MRTSYLLLFTLCLLLSEMASGGNFLTGLGHRSDHYNCVSSGGQCLYSACPIFTKIQGTCY 60
DEFB1_ptr        MRTSYLLLFTLCLLLSEMASGGNFLTGLGHRSDHYNCVSSGGQCLYSACPIFTKIQGTCY 60
DEFB1_ggo        MRTSYLLLFTLCLLLSEIASGGNFLTGLGHRSDHYNCVSSGGQCLYSACPIFTKIQGTCY 60
DEFB1_ppy        MRTSYLLLFTLCLLLSEMASGGNFLTGLGHRSDHYNCVSSGGQCLYSACPIFTKIQGTCY 60
DEFB1_hla        MRTSYLLLFTLCLLLSEMASGDNFLTGLGHRSDHYNCVRSGGQCLYSACPIYTKIQGTCY 60
DEFB1_mfa        MRTSYLLLFTLCLLLSEMASGDNFLTGLGHRSDHYNCVRSGGQCLYSACPIYTRIQGTCY 60
DEFB1_mam        MRTSYLLLFTLCLLLSEMASGDNFLTGLGHRSDHYNCVRSGGQCLYSACPIYTRIQGTCY 60
                 *****************:***.**************** ************:*:******
                                        P             P

DEFB1_hsa        RGKAKCCK 68
DEFB1_ptr        GGKAKCCK 68
DEFB1_ggo        GGKAKCCK 68
DEFB1_ppy        RGKAKCCK 68
DEFB1_hla        QGKAKCCK 68
DEFB1_mfa        HGKAKCCK 68
DEFB1_mam        HGKAKCCK 68
                  *******
                 S
```

```
DEFB4_hsa_1      MRVLYLLFSFLFIFLMPLPGVFGGIGDPVTCLKSGAICHPVFCPRRYKQIGTCGLPGTKC 60
DEFB4_ptr_1      MRVLYLLFSFLFIFLMPLPGVFGGISDPVTCLKSGAICHPVFCPRRYKQIGTCGLPGTKC 60
DEFB4_ggo_1      MRVLYLLFSFLFIFLMPLPGVFGGIGDPVTCLKSGAICHPVFCPRRYKQIGTCGLPGTKC 60
DEFB4_ppy_1      MRVLYLLFSFLFIFLMPLPGVFGGIGDPVTCLKSGAICHPVFCPRRYKQIGTCGLPGTKC 60
DEFB4_hla_1      MRVLYLLFSFPFIFLMPLPGVFGDIRNPVTCLKSGAICHPVFCPRRYKQIGTCGLPGTKC 60
DEFB4_mfa_1      MRVLYLLFSFPFIFLMPLPGVFGDIRNPVTCVRSGAICLPGFCPRRYKHIGVCGVSAIKC 60
DEFB4_mam_1      MKVLYLLFSFLFIFLMPLPGVFGDIRNPVTCVRSGAICLPGFCPRRYKHIGVCGVSAIKC 60
                 *:******** ************.* :****::***** * *******:**.**:.. **

DEFB4_hsa_1      CKKP 64
DEFB4_ptr_1      CKKP 64
DEFB4_ggo_1      CKKP 64
DEFB4_ppy_1      CKKP 64
DEFB4_hla_1      CRKP 64
DEFB4_mfa_1      CKKP 64
DEFB4_mam_1      CKKP 64
                 *:**
```

```
DEFB103_hsa_1      MRIHYLLFALLFLFLVPVPGHGGIINTLQKYYCRVRGGRCAVLSCLPKEEQIGKCSTRGR 60
DEFB103_ptr_1      MRIHYLLFALLFLFLVPVPGHGGIINTLQKYYCRVRGGRCAVLTCLPKEEQIGKCSTRGQ 60
DEFB103_ggo_1      MRIHYLLFTLLFLFLVPVPGHGGIINTLQKYYCRVRGGRCAVLSCLPKEEQIGKCSTRGR 60
DEFB103_ppy_1      MRIHYLLFALLFLFLVPVPGHGGIINTLQKYYCRVRGGRCAVLSCLPKEEQIGKCSTRGR 60
DEFB103_mam_1      MRIHYLLFALLFLFLVPVPGHGGIINTLQKYYCRVRGGRCAVLSCLPKEEQIGKCSTRGR 60
                   ********:**********************************:***************:

DEFB103_hsa_1      KCCRRKKK 68
DEFB103_ptr_1      KCCRRKKE 68
DEFB103_ggo_1      KCCRRKKK 68
DEFB103_ppy_1      KCCRRKKK 68
DEFB103_mam_1      KCCRRKKK 68
                   *******:
```

```
DEFB104_hsa_1      MQRLVLLLAISLLLYQDLPVRSEFELDRICGYGTARCRKKCRSQEYRIGRCPNTYACCLR 60
DEFB104_ptr_1      MQRLVLLLAISLLLYQDLPVRSEFELDRICGYGTARCRKKCRSQEYRIGRCPNTYACCLR 60
DEFB104_ggo_1      MRRLVLLLAISLLLYQDLPVRSEFELDRICGYGTARCRKKCRSQEYRIGRCPNTFACCLR 60
DEFB104_ppy_1      MRRLVLLLAISLLLYQDLPVRSEFELDRICGYGTARCRKKCRSQEYRIGRCPNTYACCLR 60
DEFB104_hla_1      MRRLVLLLTISVLLYQDLPVRSEFEWDRICGYGTARCRNKCRSQEYRIGRCPNTFACCLR 60
DEFB104_mam_1      MQRLVLLLAISLLFYQDLPVRSEFELDRICGYGTARCRNKCRSQEYKIGRCPNSYACCLR 60
                   *:******:**:*:*********** ************:*******:******::*****

DEFB104_hsa_1      KWDESLLNRTKP 72
DEFB104_ptr_1      KWDESLLNRTKP 72
DEFB104_ggo_1      KWDESLLNRTKP 72
DEFB104_ppy_1      KWDESLLNRTKP 72
DEFB104_hla_1      KWDESLLNSTKP 72
DEFB104_mam_1      KWDESLLNRTKP 72
                   ******** ***


DEFB105_hsa_1      MALIRKTFYFLFAMFFILVQLPSGCQAGLDFSQPFPSGEFAVCESCKLGRGKCRKECLEN 60
DEFB105_ptr_1      MALIKKTFFFLFAMFFILVQLSSGCQAGLDFSQPFPSGEFAVCESCKLGRGKCRKECLEN 60
DEFB105_ggo_1      MALIRKTFYFLFAVFFILVQLPSGCQAGLDFSQPFPSGEFAVCESCKLGRGKCRKECLEN 60
DEFB105_ppy_1      MALIRKTFYFLFAVFFVLVQLPSECQAGLDFSQPFPSDEFAVCESCKLGRGKCRKECLEN 60
DEFB105_hla_1      MALIRKTFYFLFAVFFILVQLPSGCQAGLDFSQPFPSGEFAVFESCKFSRGKCRKECLEN 60
DEFB105_mfa_1      MALIRKTFYFVFAVFFILVQQPSGCQAGLEFSEPFPSGRFAVCESCKLGRGKCRKECLEN 60
DEFB105_mam_1      MALIRKTFYFVFAVFFVLVQQPSGCQAGLEFSEPFPSGRFAVCESCKLGRGKCRKECLEN 60
                   ****:***:*:**:**:*** .* *****:**:****..*** ****:.***********

DEFB105_hsa_1      EKPDGNCRLNFLCCRQRI 78
DEFB105_ptr_1      EKPDGNCRLNFLCCRQRI 78
DEFB105_ggo_1      EKPDGNCRLNFLCCRQRI 78
DEFB105_ppy_1      EKPDGNCRLNFLCCRERI 78
DEFB105_hla_1      EKPDGNCRLNFLCCRQSI 78
DEFB105_mfa_1      EKPDGSCRLNFLCCRPRM 78
DEFB105_mam_1      ERPDGSCRLNFLCCRPRM 78
                   *:***.*********  :


DEFB106_hsa_1      MRTFLFLFAVLFFLTPAKNAFFDEKCNKLKGTCKNNCGKNEELIALCQKSLKCCRTIQPC 60
DEFB106_ptr_1      MRTFLFLFAVLFFLTPAKNAFFDEKCNKLKGTCKNNCGKNEELIALCQKSLKCCRTIQPC 60
DEFB106_ggo_1      MRTFLFLFAVLFFLTPAKNAFFDEKCNKLKGTCKNNCGKNEELIALCQKSLKCCRTIQPC 60
DEFB106_ppy_1      MRTFLFLFAVLFFLTPAKNEFFDEKCGKLKGTCKNNCGKNEELIALCQKSLKCCRTIQPC 60
DEFB106_mfa_1      MRTFLFLFVVLFFLTPAKNAFFDDKCDKLRGTCKNSCEKNEELTSFCQKSLKCCRTIQTC 60
DEFB106_mam_1      MRTFLFLFVVLFFLTPAKNAFFDDKCDKLRGTCKNNCEKNEELTSFCQKSLKCCRTIQTC 60
                   ********.********** ***:**.**:*****.* ***** ::************.*

DEFB106_hsa_1      GSIID 65
DEFB106_ptr_1      GSIID 65
DEFB106_ggo_1      GSIID 65
DEFB106_ppy_1      GSIID 65
DEFB106_mfa_1      GNTTD 65
DEFB106_mam_1      GNTTD 65
                   *.  *
```

```
DEFB107_hsa_1      MKIFVFILAALILLAQIFQARTAIHRALISKRMEGHCEAECLTFEVKIGGCRAELAPFCC 60
DEFB107_ptr_1      MKIFFFIFAALILLAQIFQARTAIHRALISKRMEGHCEAECLTFEVKTGGCRAELAPFCC 60
DEFB107_ggo_1      MKIFFFIFAALFLLAQIFQARTAIHRALICKRMEGHCEAECLTFEAKIGGCRAELAPFCC 60
DEFB107_ppy_1      MKIFFFIFAALILLAQIFQARTAIHRALICKRMEGHCEAECLTFEVKIGGCRAELAPFCC 60
DEFB107_hla_1      MKIFFFIFAALILLAQIFQARTAIHRALICKRMEGHCEAECLTFEVKIGGCRAELAPFCC 60
DEFB107_mfa_1      MKIFFFIFAALILLAQIFQARTAIHRALICKRMEGHCEAECLTFEVKIGGCRAELTPYCC 60
DEFB107_mam_1      MKIFFFIFAALILLAQIFQARTAIHRALICKRMEGHCEAECLTFEVKIGGCRAELTPYCC 60
                   ****.**:***:*****************.***************.* *******:*:**

DEFB107_hsa_1      KNRKKH 66
DEFB107_ptr_1      KNRKKH 66
DEFB107_ggo_1      KNRKKH 66
DEFB107_ppy_1      KNRKKH 66
DEFB107_hla_1      KNRKKH 66
DEFB107_mfa_1      KKRKKD 66
DEFB107_mam_1      KKRKKD 66
                   *:***.
```

```
DEFB118_hsa      MKLLLLALPMLVLLPQVIPAYSGEKKCWNRSGHCRKQCKDGEAVKDTCKNLRACCIPSNE 60
DEFB118_ptr      MKLLLLALPVLVLLPQVIPAYSGEKKCWNRSGHCRKQCKDGEAVKDTCKNLRACCVPSNE 60
DEFB118_ggo      MKLLLLALPMLVLLPQVIPAYSGEKKCWNRSGHCRKQCKDGEAVKDTCKNLRACCVPSNE 60
DEFB118_ppy      MKLLLLALPMLVLLPQVIPAYSGEKKCWNRSGHCRKQCKDGEAVKDTCKNLRACCVPSNE 60
DEFB118_hla      MKLLLLALPMLVLLPQVIPAYSGEKKCWNRSGHCRKQCKDGEAVKDTCKNLRACCVPSNE 60
DEFB118_mfa      MKLLLLALPILVLLPQVIPAYGGEKKCWNRSGHCRKQCKDGEAVKETCKNHRACCVPSNE 60
DEFB118_mam      MKLLLLALPILVLLPQVIPAYGGEKKCWNRSGHCRKQCKDGEAVKETCKNHRACCVPSNE 60
                 *********:***********.***********************:**** ****:****
                                                  P                     P

DEFB118_hsa      DHRRVPATSPTPLSDSTPGIIDDILTVRFTTDYFEVSSKKDMVEESEAGRGTETSLPNVH 120
DEFB118_ptr      DHRRVPMTSPTPLSDSTPGIIDDILTVRFTTDYFEVSSKKDMVEESEAGRGTETSLPNVH 120
DEFB118_ggo      DHRRVPTTSPTPLSDSTPGIIDDILTVRFTTDYFEVSSKKDMIEESEAGRGTETSLPNVH 120
DEFB118_ppy      DHRRVPTTSPTPLSDSTRGVIDDILTVRFTTDYFEVSSKKNMVEESEVGQGTQTSLPNVH 120
DEFB118_hla      DHRQVPTTSPTPLSDSTPGSIDDILTVRFTTDYFEVSSKKDMVEESEAGWGTQTSLPDVH 120
DEFB118_mfa      DHRRVPTTSPTPLSDSTPGIIDNILTIRFTTDYFEISSKKDMVEESEAGQGTQTSPPNVH 120
DEFB118_mam      DHRRLPTTSPTPLSDSTPGIIDNILTIRFTTDYFEISSKKDMVEESEAGQGTQTSPPNVH 120
                 ***::* ********** * **:***:********:****:*:****.* **:** *:**
                                                                  S

DEFB118_hsa      HSS 123
DEFB118_ptr      HSS 123
DEFB118_ggo      HSS 123
DEFB118_ppy      HSS 123
DEFB118_hla      HSS 123
DEFB118_mfa      HTS 123
DEFB118_mam      HTS 123
                 *:*
```

```
DEFB119_hsa_1       MKLLYLFLAILLAIEEPVISGKRHILRCMGNSGICRASCKKNEQPYLYCRNCQSCCLQSY 60
DEFB119_ptr_1       MKLLYLFLAILLAIEEPVISGKRHILRRMGNSGICRASCKKNEQPYLYCRNYQSCCLQSY 60
DEFB119_ggo_1       MKLLYLFLAILLAIEEPVISGKRHILRCMGNSGICRASCKKNEQPYLYCRNYQSCCLQSY 60
DEFB119_ppy_1       MKLLYLFLAILLVIEEPVISGKRYILRCMGNSGICRASCKRNEQPYLYCKNYQSCCLQSY 60
DEFB119_hla_1       MKLLYLFLAILLAIEEPVISGKHHILRCMGNSGICRASCKKNEQPYLYCRNYQHCCLQSY 60
DEFB119_mfa_1       MKFLFLFLAILLATKIPVISGKRHNLRCMGNSGICRASCKKNEQPYLYCRNYQACCLQSY 60
DEFB119_mam_1       MKFLFLFLAILLATEVPVISGKRHILRCMGNSGICRASCKKNEQPYLYCRNYQACCLQSY 60
                    **:*:*******. : ******:: ** ************:********:* * ******

DEFB119_hsa_1       MRISISGKEENTDWSYEKQWPRLP 84
DEFB119_ptr_1       MRISISGKEENTDWSYEKQWPRLP 84
DEFB119_ggo_1       MRISISGKEEDTDWSYEKQWPRLP 84
DEFB119_ppy_1       MRISISGKEENTDWSYEKQWPKLP 84
DEFB119_hla_1       MRISISGEEENTDWSYEKQWPRLP 84
DEFB119_mfa_1       MRISISGKEENTDWSYEKQWPRLP 84
DEFB119_mam_1       MRISISGKEENTDWSYEKQWPRLP 84
                    *******:**:**********:**


DEFB120_hsa_1       MKLLYLFLAILLAIEEPVISVECWMDGHCRLLCKDGEDSIIRCRNRKRCCVPSRYLTIQP 60
DEFB120_ptr_1       MKLLYLFLAILLAIEEPVISVECWMDGHCRLLCKDGEDSIIRCRNRKRCCVPSRYLTIQP 60
DEFB120_ggo_1       MKLLYLFLAILLAIEEPVISVECWMDGHCRLLCKDGEDSIIRCRNRKRCCVPSHYLTIQP 60
DEFB120_ppy_1       MKLLYLFLAILLVIEEPVISVECWMDGHCRLLCKDGEDSIIRCRNRKRCCVPSHYLTIQP 60
DEFB120_hla_1       MKLLYLFLAILLAIEEPVISVECWMDGHCRLLCKDGEDSIIRCRNRKRCCVPSRYLTIQP 60
DEFB120_mfa_1       MKFLFLFLAILLATKIPVISVECWMDGHCRLLCKDGEDSIIRCRNRKRCCVPSRYLTIQP 60
DEFB120_mam_1       MKFLFLFLAILLATEVPVISVECWMDGHCRLLCKDGEDSIIRCRNRKRCCVPSRYLTIQP 60
                    **:*:*******. : *************************************:******
                                               P                 P

DEFB120_hsa_1       VTIHGILGWTTPQMSTTAPKMKTNITNR 88
DEFB120_ptr_1       VTIHGILGWTTPQMSTTAPKTKTNITNR 88
DEFB120_ggo_1       VTIHGILGWTTPQMSTTAPKTKTNITNR 88
DEFB120_ppy_1       VTIHGILGWTTPQMPTTAPKAKRNITNR 88
DEFB120_hla_1       VTIHGILGWTTPQMSTTAPKMKRNITNR 88
DEFB120_mfa_1       VTIHGILGWTTPRMSTTAPQPKRNIHNG 88
DEFB120_mam_1       VTIHGILGWTTPRMSTTAPQPKRNIHNG 88
                    ************:*.****: * ** *
                                        S


DEFB123_hsa_1       MKLLLLTLTVLLLLSQLTPGGTQRCWNLYGKCRYRCSKKERVYVYCINNKMCCVKPKYQP 60
DEFB123_ptr_1       MKLLLLTLTVLLLLSQLTPGGTQRCWNLYGKCRYRCSKKERVYVYCINNKMCCVKPKYQP 60
DEFB123_ggo_1       MKLLLLTLTVLLLLSQLTPGGTQRCWNLYGKCRYRCSKKERVYVYCINNKMCCVKPKYQP 60
DEFB123_ppy_1       MKLLLLTLTVLLLLSQLTPGGTQRCWNLYGKCRHRCSKKERVYVYCVNNKMCCVKPKYQP 60
                    *********************************:************:*************

DEFB123_hsa_1       KERWWPF 67
DEFB123_ptr_1       KERWWPF 67
DEFB123_ggo_1       KERWWPF 67
DEFB123_ppy_1       KERWWRF 67
                    ***** *
```

```
DEFB125_hsa      MNILMLTFIICGLLTRVTKGSFEPQKCWKNNVGHCRRRCLDTERYILLCRNKLSCCIS-I 59
DEFB125_ptr      MNILMLTFIICGLLTQVTKGSFEPPKCGNNNIGHCRKRCLDTERYILLCRNKLSCCIYII 60
DEFB125_ggo      MNILMLTFIICGLLTQVTKGSFEPQKCWKNNIGHCRRRCLDTERCILLCRNKLSCCIYII 60
DEFB125_ppy      MNLLMLTFIICGLLTQVTKGSFEPQKCWKNNIGHCRRRCLDTERYILLCRNKLSCCISII 60
DEFB125_hla      MNLLMLTFIICGLLTQVTKGSFEPPKCWKNNIGHCRRRCLDTERYILVCRNKLSCCISII 60
DEFB125_mfa      MNLLILTFIICGLLTQVTKGSFEPQKCWKNNIGYCRRRCLDTERYILLCRNKLSCCISII 60
DEFB125_mam      MNLLILTFIICGLLTQVTKGSFEPQKCWKNNIGYCRRRCLDTERYILLCRNKLSCCISII 60
                 **:*:**********:******** ** :**:*:**:******* **:**********

DEFB125_hsa      ISHEYTRRPAFPVIHLEDITLDYSDVDSFTGSPVSMLNDLITFDTTKFGETMTPETNTPE 119
DEFB125_ptr      ISHEYTRRPAFPMIHLEDITFDYSDVDSFTGSPVSMLNDLITFDTTKFGETMTPETNTPE 120
DEFB125_ggo      ISHEYTQRPAFPMIHLEDITFDYSDVDSFTGSPVSMLNDLITFDTTKFGETMTPETNTPE 120
DEFB125_ppy      ISHEYTRRPAFPVIHLEDITFDYSDVDSFTGSPVSMLNDLITFDTTKFGETITPETNTPE 120
DEFB125_hla      ISHEYTRRPAFPVIHPEDITFDYSDVDSFTGSPVSMLNDLITFDTTKFGETMTPETNTPE 120
DEFB125_mfa      ISYEYTRRPPFRVIHLEDITFDYSDMGSFTGSPVSKLSDLVTFDIRR--ETMTPKTNTPE 118
DEFB125_mam      ISYEYTRRPPFRVIHLEDITFDYSDMGSFTGSPVSKLSDLVTFDIRR--ETMTPKTNTPE 118
                 **:***:**.* :** ****:****:.******** *.**:***  :  **:**:*****

DEFB125_hsa      TTMPPSEATTPETTMPPSETATPRLCHHLLRQLLLIIN 157
DEFB125_ptr      TTMPPSETTTPETTMPPSETATSETMPPLS-QTALTHN 157
DEFB125_ggo      TTVPPSETTTPETTMPPSETATSETMPPPS-QTALTHN 157
DEFB125_ppy      TTMPPSETTSSKTTMPPSETATSET-MPPPSQTALTHN 157
DEFB125_hla      TTMPPS------------KTTTSKTTMPPPSQTALTHN 146
DEFB125_mfa      TTMPPSETTTSKTTMPSSKTTTSKTTMPPPSQMALTHN 156
DEFB125_mam      TTMPPSETTTSKTTMPSSKTTTSKTTMPPPSQMALTHN 156
                 **:***            :*:*..       *  *  *


DEFB126_hsa      MKSLLFTLAVFMLLAQLVSGNWYVKKCLNDVGICKKKCKPEEMHVKNGWAMCGKQRDCCV 60
DEFB126_ptr      MKSLLFTLAVFMLLAQLVSGNWYVKKCLNDVGICKKKCKPGEMHIKNGWATCGKQRDCCV 60
DEFB126_ggo      MKSLLFTLAVFMLLAQLVSGNWYVKKCLNDVGICKKKCKPEEMHVKNGWAMCGKQRDCCV 60
DEFB126_ppy      MKSLLFTLAVFMLLAQLVSGSWYVKKCLNDVGICKKKCKPEELHVKNGWAMCGKQRDCCV 60
DEFB126_hla      MKSLLFTLAVFMLLAQLVSGNWYVKKCLNDVGICKKKCKPEELHVKNGRAMCGKQRDCCV 60
DEFB126_mfa      MKSLLFTLAVFMLLAQLVSGNLYVKRCLNDIGICKKTCKPEEVRSEHGWVMCGKRKACCV 60
DEFB126_mam      MKSLLFTLAVFMLLAQLVSGNLYVKRCLNDIGICKKTCKPEEVRSEHGWVMCGKRKACCV 60
                 ********************. ***:****:*****.*** *:: ::* . ***:: ***

DEFB126_hsa      PADRRANYPVFCVQTKTTRISTVTATTAT---------TTLMMTTASMSSMA-------- 103
DEFB126_ptr      PADRRANYPAFCVQTKTTRTSTVTAK------------TTLMVTTASMSSMA-------- 100
DEFB126_ggo      PADRRANYPAFCVQTKTTRTSTVTATTAT---------TTLMMTTASMSLMA-------- 103
DEFB126_ppy      PADKRANYPAFCVQTKTTRTSTVTATTATRATTAT--TTTLMMTTASMSSMT-------- 110
DEFB126_hla      PADKRANYPAFCVQTKTTRTSTVTATAAT--------TTTLVMTTASMSSMA-------- 104
DEFB126_mfa      PADKRSAYPSFCVHSKTTKTSTVTARATATTATTATAATPLMISNGLISLMSYDGRYPCF 120
DEFB126_mam      PADKRSAYPSFCVRSKTTKTSTVTARATATTATTATAATPLMISNGLISLMT-------- 112
                 ***:*: ** ***::***: *****             *.*:::.. :* *:

DEFB126_hsa      ----PTRFSHWLSLCLLLX 118
DEFB126_ptr      ----PTPVSPTG------- 108
DEFB126_ggo      ----PTPVSPTG------- 111
DEFB126_ppy      ----PTPVSPTG------- 118
DEFB126_hla      ----PTPVSPTS------- 112
DEFB126_mfa      SHYLNIPASVSCSRS---- 135
DEFB126_mam      ----TMAATPVSPTT---- 123
```

```
DEFB127_hsa_1       MGLFMIIAILLFQKPTVTEQLKKCWNNYVQRHCRKICRVNEVPEALCENGRYCCLNIKEL 60
DEFB127_ptr_1       MGLFMIIAILLFQKPTVTEQLKKCWNNYVQGHCRKICRVNEVPEALCENGRYCCLNIKEL 60
DEFB127_ggo_1       MGLFMIIAILLFQKPTVTEQLKKCWNNYVQGHCRKICRVNELPEALCENGRYCCLNIKEL 60
DEFB127_ppy_1       MGLFMIIAILLFQKPTVTEQLKKCWNNYVQGHCRKICRINEVREALCENGRYCCLNINEL 60
DEFB127_hla_1       MGLFMIIAVLLFQKPTVTEQLKKCWDNYVQGRCRKICRVNEVREALCEDGRYCCLNIKEL 60
DEFB127_mfa_1       ----MIIVILLFQKPTVTEQLKTCWDNYVQGHCRKICRVN----ALCENGRYCCLNIKEL 52
DEFB127_mam_1       ----MIIVILLFQKPTVTEQLKTCWDNCVQGHCRKICRVN----ALCENGRYCCLNIKEL 52
                        ***.:*************.**:* ** :******:*    ****:********:**
                                                  P                          P

DEFB127_hsa_1       EACKKITKPPSPKPATLALTL-QDYVTIIENFPSL 94
DEFB127_ptr_1       EACKKITKPSHPKPATLALTL-QDYVTIIENFPSL 94
DEFB127_ggo_1       EACKKITKPPRPKPATLALTL-QDYVTIIENFPSL 94
DEFB127_ppy_1       EACKKITKPPHPKPATFALTLPQDYVTIIENFPSL 95
DEFB127_hla_1       EACKKITKPPRPKPATLALTLPQDYVTIIENFPIL 95
DEFB127_mfa_1       EACKKITNSPHPKPATLALTLPQDYVTITEKFPIL 87
DEFB127_mam_1       EACKKITNSPHPKPATLALTLPQDYVTITEKFPIL 87
                    *******:.. *****:**** ****** *:** *
                              P
                              S

DEFB128_hsa_1       MKLFLVLIILLFEVLTDGARLKKCFNKVTGYCRKKCKVGERYEIGCLSGKLCCANDEEEK 60
DEFB128_ggo_1       MKLFLVLIILLFEVLTDGARLKKCFNNVTGYCRKKCKVGERYEIGCLSGKLRCANDEEEK 60
DEFB128_ppy_1       MKLFLVLIILLFEVLTDGARLKKCFNNVTGYCRKKCKVGERYEIGCLSGKLCCINYEEEK 60
DEFB128_hla_1       MKLFLVLIILLFEVLTDGARLKKCFNNITGYCRKKCKVGERYEIGCLSGKLCCVNDEEEK 60
DEFB128_mfa_1       MKLFLVLIILLFEVLTDGARLKKCFNNVTGYCRKKCKVGEIHEIGCLSGKLCCVNDEENK 60
DEFB128_mam_1       MKLFLVLIILLFEVLTDGARLKKCFNNVTGYCRKKCKVGEIHEIGCLSGKLCCVNDEENK 60
                    **************************::************ :********* * * **:*

DEFB128_hsa_1       KHVSFKKPHQHSGEKLSVLLDYIILPTITIFTV 93
DEFB128_ggo_1       KRVI----------------------------- 64
DEFB128_ppy_1       EHVSFKKPHQHSGEKLSVQQDYIILPTVTIFTV 93
DEFB128_hla_1       KHVSFKKPHQHSGEKLSVQQDYIILPTITIFAV 93
DEFB128_mfa_1       KHVPFKKPHQQPVEKLSVQQDYVILPTITIFTV 93
DEFB128_mam_1       KHVPFKKPHQQPVEKLSVQQDYVILPTITIFTV 93
                    ::*
```

```
DEFB129_hsa      MKLLFPIFASLMLQYQVNTEFIGLRRCLMGLGRCRDHCNVDEKEIQKCKMKKCCVGPKVV 60
DEFB129_ptr      MKLLFPIFASLMLQYQVNTEFIGLRRCLMGLGRCRDHCNVDEKEIQKCKMKKCCVGPKVV 60
DEFB129_ggo      MKLLFPIFASLMLQYQVNTEFIGLRRCLMGLGRCRDHCNVDEKEIQKCKMKKCCVGPKVV 60
DEFB129_ppy      MKLLFPVFASLMLQYQVNTEFIGLRRCLMGFGRCRDHCNVDEKEIQKCKMKKCCVGPKVV 60
DEFB129_hla      MKLLFPIFASLMLQYKVNTEFIGLRRCLMGFGRCRDHCNVDEKEIQKCKMKKCCVGPKVV 60
DEFB129_mfa      MKLLFPIFASLMLQYQVNTEFIGLRSCLMGFGRCRDHCNVDEKEIQKCKMKKCCVGPKVV 60
DEFB129_mam      MKLLFPIFASLMLQYQVNTEFIGLRSCLMGFGRCRDHCNVDEKEIQKCKMKKCCVGPKVV 60
                 ******:********:********* ****:*****************************

DEFB129_hsa      KLIKNYLQYGTPNVLNEDVQEMLKPAKNSSAVIQRKHILSVLPQIKSTSFFANTNFVIIP 120
DEFB129_ptr      KLIKNYLQYGTPNVLNEDVQEMLKPAKNSSAVIQRKHILSVLPQIKSTSFFANTNFVIIP 120
DEFB129_ggo      KLIKNYLQYGTPNVLNEDVQEMLKPAKNSSAVIQRKHILSVLPQIKSTSFFANTNFVIIP 120
DEFB129_ppy      KLIKNYLQYGIPNVLNEDVQEMLKPAKNSSAVIQRKHILSVLPQIKSTSFFANTNFVIIP 120
DEFB129_hla      KLIKNYLQYGTPNVLNEDVQEMLKSAKNSSAVIQRKHILSVLPQIKSTSFFANTNFVIIP 120
DEFB129_mfa      KLIKNYLQYGTPNVLNEDVQEMLKPAENSSAVIQRKHILSILPQIKSINFFANTNLVIIP 120
DEFB129_mam      KLIKNYLQYGTPNVLNEDVQEMLKPAENSSAVIQRKHILSILPQIKSINFFANTNLVIIP 120
                   ********** *************.*:*************:****** .******:****

DEFB129_hsa      NATPMNSATISTMTPGQITYTATSTKSNTKESRDSATASPPPAPPPPNILPTPSLELEEA 180
DEFB129_ptr      NATPMNSATISTMTPGQITYTATSTKSNTKESRDSATASPPPAPPPPNILPTPSLELEEA 180
DEFB129_ggo      NATPMNSATISTMTPGQITYTATSTKSNTKESRDSATASSPPAPPPPNILPTPSLELEEA 180
DEFB129_ppy      NATPMNSATISTMTPGQITYTATSTKSNTKESRDSATASPPPAPPPPNILPTPSLELEKA 180
DEFB129_hla      NATPMNSAIISTVTPGQITYTAASTKSNIKESRDSATASPPPAPPPPNTLPTP------- 173
DEFB129_mfa      NATPVNSATVSTMTSGQITYTATSAKSNTKESGDSATASPPPAPPPPNILPTPSLELEEA 180
DEFB129_mam      NATPVNSATVSTMTSGQITYTATSTKSNTKESRDSATASPPPAPPPPNILPTPSLELEEA 180
                 ****:*** :**:*.*******:*:*** *** ******.******** ****

DEFB129_hsa      EEQ 183
DEFB129_ptr      EEQ 183
DEFB129_ggo      EEQ 183
DEFB129_ppy      EEQ 183
DEFB129_hla      ---
DEFB129_mfa      EEQ 183
DEFB129_mam      EEQ 183
```

```
DEFB132_hsa_1      MKFLLLVLAALGFLTQVIPASAGGSKCVSNTPGYCRTCCHWGETALFMCNASRKCCISYS 60
DEFB132_ptr_1      MNFLLLVLAALGFLTQVIPASAGGSKCVSNTPGYCRTYCHWGETALFMCNASRKCCVSYS 60
DEFB132_ggo_1      MKFLLLVLAALGFLTQVIPASAGGSKCVSNTPAYCRTYCHWGETALFMFNASRKCCISYS 60
DEFB132_ppy_1      MKFLLLVLAALRFLTQVIPASAGGSKCVSNTPGYCRTYCHQGETALFMCNASRKCCVSYS 60
DEFB132_hla_1      MKFLLLVLAALRFLTQVIPASGGGSKCVSDTQGYCRTYCHQGETALFMCNASRKCCASYS 60
DEFB132_mfa_1      MKFLLLVLAALGFLTQVIPASGGGSKCVSDTPGYCRTHCHRGETALFMCSPFRKCCISYS 60
DEFB132_mam_1      MKFLLLVLAALGFLTQVIPASGGGSKCVSDTPGYCRTHCHRGETALFMCSPFRKCCISYS 60
                   *:********* *********.*******:* .**** ** ******* .. **** ***
                                                           P               S

DEFB132_hsa_1      FLPKPDLPQLIGNHWQSRRRNTQRKDKKQQTTVTS- 95
DEFB132_ptr_1      FLPKADLPRLIGNHWQSRRRNTQRKDKKQQTTVTS- 95
DEFB132_ggo_1      FLPKPDLPQLIGNHWQSRR-NTQRKDKKQQTTVTS- 94
DEFB132_ppy_1      FLPKPDLPQLIGNHWQSRRRNTQRKDKKQQTTVTS- 95
DEFB132_hla_1      FLPKPDLPQLIGNHWQSRRRNTQRKDKKQQTTVTSS 96
DEFB132_mfa_1      FLPQPDLPQLIGNHWPSRSRNTQRKNKKQQTTVTP- 95
DEFB132_mam_1      FLPQPDLPQLIGNHWPSRSRNTQRKNKKQQTTVTP- 95
                   ***:.***:****** **  *****:********.
                     P   P                         P
```
