# Supplementary material for: Directional and balancing selection in human beta-defensins
Source: BMC Evol Biol. 2008 Apr 16;8:113. doi: 10.1186/1471-2148-8-113 (PMC2373304; doi:10.1186/1471-2148-8-113)
Supplement: Additional file 2 — Supplementary Table 1 Accession numbers of primate defensin sequences. Refererence cited in the table is [32]. [file 1471-2148-8-113-S2.doc]

Supplementary table 1 – Accession numbers of primate defensin sequences

* from ref 32.

|  | *P.troglodytes* | *G. gorilla* | *P.pygmaeus* | *H. lar* | *M.fascicularis* |
| --- | --- | --- | --- | --- | --- |
| DEFB1 | AM410097 | AM410098 | AM410099 | AM410100 | AM410101 |
| DEFB4 | NM_001009076 | * | AM410102 | * | * |
| DEFB103 | AM410103 | AM410104 | AM410105 | - | - |
| DEFB104 | AM410106 | AM410107 | AM410108 | AM410109 | - |
| DEFB105 | AM410110 | AM410111 | AM410112 | AM410113 | AM410114 |
| DEFB106 | AM410115 | AM410116 | AM410117 | AM410118 | - |
| DEFB107 | AM410119 | AM410120 | AM410121 | AM410122 | AM410123 |
| DEFB118 | AM410124 | AM410125 | AM410126 | AM410127 | AM410128 |
| DEFB119 | AM410129 | AM410130 | AM410131 | AM410132 | AM410133 |
| DEFB120 | AM410134 | AM410135 | AM410136 | AM410137 | AM410138 |
| DEFB123 | AM410139 | AM410140 | AM410141 | - | - |
| DEFB125 | AM410142 | AM410143 | AM410144 | AM410145 | AM410146 |
| DEFB126 | AM410147 | AM410148 | AM410149 | AM410150 | AM410151 |
| DEFB127 | AM410152 | AM410153 | AM410154 | AM410155 | AM410156 |
| DEFB128 | - | AM410157 | AM410158 | AM410159 | AM410160 |
| DEFB129 | AM410161 | AM410162 | AM410163 | AM410164 | AM410165 |
| DEFB132 | AM410166 | AM410167 | AM410168 | AM410169 | AM410170 |
