# Supplementary material for: Directional and balancing selection in human beta-defensins
Source: BMC Evol Biol. 2008 Apr 16;8:113. doi: 10.1186/1471-2148-8-113 (PMC2373304; doi:10.1186/1471-2148-8-113)
Supplement: Additional file 3 — Supplementary Table 2 MAPH probes designed for copy number analysis. References cited in the table are [43,18,19,58]. [file 1471-2148-8-113-S3.doc]

Supplementary table 2

MAPH probes designed for copy number analysis

| Name | Type/region | Start coordinate (NCBI build 36.1) | End coordinate (NCBI build 36.1) | Reference | r^2^ |
| --- | --- | --- | --- | --- | --- |
| G13705 | 8p23 CNV | Chr8:7305559* | Chr8:7305765* | 18 | 0.96 |
| DEFB106 | 8p23 CNV | Chr8:7327364* | Chr8:7327683* | 19 | 0.87 |
| DEFB104 | 8p23 CNV | Chr8:7315241* | Chr8:7315552* | 19 | 0.86 |
| DEFB103 | 8p23 CNV | Chr8:7275181* | Chr8:7275317* | 18 | 0.84 |
| DEFB105 | 8p23 CNV | Chr8:7334310* | Chr8:7334452* | 19 | 0.83 |
| DEFB4B | 8p23 CNV | Chr8:7259889* | Chr8:7260004* | 18 | 0.71 |
| ST11G1 | reference | Chr19:63775244 | Chr19:63775469 | 43 | 0.68 |
| PX3F11 | X-linked | chrX:32547543 | ChrX:32547885 | this paper | 0.61 |
| nDLC1 | reference | Chr8:13023765 | Chr8:13023982 | 18 | 0.57 |
| DEFB126 | 20p13 | Chr20:74273 | Chr20:74405 | this paper | 0.52 |
| ST18A2 | reference | Chr2:299006 | Chr2:299246 | 43 | 0.47 |
| ANGPT2 | reference | Chr8:6347970 | Chr8:6348154 | 18 | 0.47 |
| DEFB125i | 20p13 | Chr20:25025 | Chr20:25221 | this paper | 0.46 |
| DEFB118 | 20q11 | Chr20:29424518 | Chr20:29424908 | this paper | 0.45 |
| DEFB123 | 20q11 | Chr20:29501452 | Chr20:29501707 | this paper | 0.42 |
| ST14A2 | reference | Chr4:99218 | Chr4:99638 | 43 | 0.42 |
| ST18F2 | reference | Chr7:158816019 | Chr7:158816312 | 43 | 0.33 |
| ST18C1 | reference | Chr9:352611 | Chr9:352841 | 43 | 0.32 |
| DEFB122 | 20q11 | Chr20:29472910 | Chr20:29473174 | this paper | 0.31 |
| DEFB124 | 20q11 | Chr20:29516968 | Chr20:29517163 | this paper | 0.31 |
| DEFB119 | 20q11 | Chr20:29428548 | Chr20:29428988 | this paper | 0.30 |
| DEFB114 | 6p12 | Chr6:50035885 | Chr6:50036185 | this paper | 0.29 |
| SPLUNC2 | reference | Chr20:31231963 | Chr20:31232057 | this paper | 0.28 |
| ST17A1 | reference | Chr20:247074529 | Chr20:247074685 | 43 | 0.28 |
| AA01 | reference | Chr8:8722885 | Chr8:8723076 | 18 | 0.27 |
| DEFB129 | 20p13 | Chr20:158299 | Chr20:158464 | this paper | 0.27 |
| DEFB112 | 6p12 | Chr6:50119215 | Chr6:50119522 | this paper | 0.25 |
| BPIL3 | reference | Chr20:31090695 | Chr20:31091167 | this paper | 0.25 |
| LPLUNC1 | reference | Chr20:31357427 | Chr20:31357662 | this paper | 0.23 |
| GAS11 | reference | Chr8:6602196 | Chr8:6602305 | 18 | 0.23 |
| ST4A4 | reference | Chr2:242496492 | Chr2:242496668 | 43 | 0.21 |
| LPLUNC4 | reference | Chr20:31143822 | Chr20:31144198 | this paper | 0.17 |
| ST18G1 | reference | Chr12:132169641 | Chr12:132169740 | 43 | 0.16 |
| DEFB120 | 20q11 | Chr20:29440386 | Chr20:29440815 | this paper | 0.12 |
| MCM6 | reference | Chr2:136314312 | Chr2:136314839 | this paper | 0.12 |
| LPLUNC3 | reference | Chr20:31115307 | Chr20:31115023 | this paper | 0.12 |
| TBP | reference | Chr6:170706114 | Chr6:170706362 | 58 | 0.06 |
| GATA4 | reference | Chr8:11653239 | Chr8:11653641 | 18 | 0.05 |
| ST19F2 | reference | Chr5:180620496 | Chr5:180620665 | 43 | 0.03 |
| ST8D2 | reference | Chr19:291949 | Chr19:292411 | 43 | 0.03 |
| ST19H1 | reference | Chr15:100043567 | Chr15:100043934 | 43 | 0.02 |
| ST9A3 | reference | Chr22:49488550 | Chr22:49488897 | 43 | 0.01 |
| SPLUNC1 | reference | Chr20:31294502 | Chr20:31295012 | this paper | -0.01 |
| BPI | reference | Chr20:36373657 | Chr20:36373761 | this paper | -0.01 |
| ST7B2 | reference | Chr16:18855* | Chr16:19224* | 43 | -0.03 |
| F11 | reference | Chr4:187424329 | Chr4:187424436 | 58 | -0.08 |
| ST11G9 | reference | Chr19:63772964 | Chr19:63773090 | 43 | -0.08 |
| LBP | reference | Chr20:36408444 | Chr20:36408360 | this paper | -0.08 |
| BPIL1 | reference | Chr20:31070028 | Chr20:31070365 | this paper | -0.11 |

n.b Defensin genes not described in the main paper but probes described here did not show evidence of full-length transcripts at the start of the project. None of these probes showed evidence of copy number variation.

* full length of probe maps to two positions in this assembly
