# Supplementary material for: Directional and balancing selection in human beta-defensins
Source: BMC Evol Biol. 2008 Apr 16;8:113. doi: 10.1186/1471-2148-8-113 (PMC2373304; doi:10.1186/1471-2148-8-113)
Supplement: Additional file 4 — Supplementary Table 3 DEFB132 haplotypes. [file 1471-2148-8-113-S4.doc]

Supplementary table 2 – *DEFB132* haplotypes

| Site number | 1 | 2 | 3 | 4 | 5 | 6 | 7 | 8 | 9 | 10 |
| --- | --- | --- | --- | --- | --- | --- | --- | --- | --- | --- |
| Build35 –chr20 | 187664 | 187688 | 187697 | 187749 | 187781 | 187847 | 187867 | 187881 | 187936 | 187937 |
| rs number | 420525 | 399083 | 399094 | - | - | - | - | - | - | - |
| NA02476 (SSA) | C | A | C | C | G | C | C | C | G | T |
|  | T | G | G | C | C | C | T | C | G | T |
| NA08688 (SSA) | C | A | C | C | G | C | C | C | G | T |
|  | C | A | C | C | G | C | C | C | G | T |
| HAM001 (SSA) | C | A | C | C | G | A | C | C | G | T |
|  | C | G | C | C | G | C | C | T | G | C |
| HAM006 (SSA) | C | A | C | C | G | C | C | C | G | T |
|  | T | G | G | C | G | C | C | C | G | T |
| HAM008 (SSA) | C | A | C | C | G | C | C | C | G | T |
|  | T | G | G | C | G | C | C | C | G | T |
| NA02064 (SSA) | C | A | C | C | G | C | C | C | G | T |
|  | C | A | C | C | G | C | C | C | G | T |
| NA00522 (SSA) | C | A | C | C | G | A | C | C | G | T |
|  | C | A | C | C | G | A | C | C | G | T |
| IHLAD031 (IA) | C | A | C | C | G | C | C | C | G | T |
|  | C | A | C | C | G | C | C | C | G | T |
| BUR-E (IA) | C | A | C | C | G | C | C | C | A | T |
|  | C | A | C | C | G | C | C | C | G | T |
| WOO-EM (IA) | C | A | C | C | G | C | C | C | G | T |
|  | C | A | C | C | G | C | C | C | G | T |
| HAY-BD (IA) | T | G | G | C | G | C | C | C | G | T |
|  | T | G | G | C | G | C | C | C | G | T |
| IHLAD036 (IA) | C | G | G | C | G | C | C | C | A | C |
|  | C | G | G | C | G | C | C | C | G | T |
| NON-L (IA) | C | A | C | C | G | C | C | C | G | T |
|  | T | A | C | C | G | C | C | C | G | T |
| WON-M (IA) | C | A | C | C | G | C | C | C | G | T |
|  | T | G | G | C | G | C | C | C | A | C |
| WON-I (IA) | C | A | C | C | G | C | C | C | G | T |
|  | C | A | C | C | G | C | C | C | G | T |
| WON-C (IA) | C | A | C | C | G | C | C | C | G | T |
|  | T | G | G | C | G | C | C | C | A | C |
| HAY-KJ (IA) | C | A | C | C | G | C | C | C | G | T |
|  | T | G | G | C | G | C | C | C | G | T |
| NA10469 (MB) | C | G | C | C | G | C | C | C | G | T |
|  | C | G | C | C | G | C | C | C | G | T |
| NA10470 (MB) | C | A | C | C | G | C | C | C | G | T |
|  | C | G | C | C | G | C | C | T | G | C |
| NA10471 (MB) | C | A | C | C | G | C | C | C | G | T |
|  | C | A | C | C | G | A | C | C | G | T |
| NA10472 (MB) | C | A | C | C | G | C | C | C | G | T |
|  | C | A | C | C | G | C | C | C | G | T |
| NA10473 (MB) | C | A | C | C | G | C | C | C | G | T |
|  | C | G | C | C | G | C | C | C | G | T |
| NA10492 (MB) | C | G | C | C | G | C | C | T | G | C |
|  | T | G | G | C | G | C | C | C | A | C |
| NA10493 (MB) | C | A | C | C | G | C | C | C | G | T |
|  | T | G | G | C | G | C | C | C | A | C |
| NA10494 (MB) | C | G | G | C | G | C | C | T | G | C |
|  | C | G | G | C | G | C | C | T | G | C |
| NA10495 (MB) | T | G | G | C | C | C | T | C | G | T |
|  | T | G | G | T | G | C | C | C | G | T |
| NA10496 (MB) | C | A | G | C | G | C | C | T | G | C |
|  | C | G | G | C | G | C | C | T | G | C |
| CO038 (UK) | C | A | C | C | G | C | C | C | G | T |
|  | C | A | C | C | G | C | C | C | G | T |
| CO156 (UK) | T | G | G | C | G | C | C | C | G | T |
|  | T | G | G | T | G | C | C | C | G | T |
| CO157 (UK) | C | A | C | C | G | C | C | C | G | T |
|  | C | A | C | C | G | C | C | C | G | T |
| CO744 (UK) | C | A | C | C | G | A | C | C | G | T |
|  | C | G | C | C | G | C | C | T | G | C |
| CO896 (UK) | T | G | G | C | G | C | C | C | G | T |
|  | T | G | G | C | G | C | C | C | G | T |
| CO917 (UK) | T | G | G | C | G | C | C | C | G | T |
|  | T | G | G | C | G | C | C | C | G | T |
| CO183 (UK) | T | G | G | C | G | C | C | C | G | T |
|  | T | G | G | C | G | C | C | C | G | T |
| CO152 (UK) | C | A | C | C | G | C | C | C | G | T |
|  | T | G | G | C | G | C | C | C | G | T |
| CO208 (UK) | C | A | C | C | G | C | C | C | G | T |
|  | T | G | G | C | G | C | C | C | G | T |
| CO187 (UK) | C | A | C | C | G | C | C | C | G | T |
|  | T | G | G | C | G | C | C | C | G | T |
