# Supplementary material for: Directional and balancing selection in human beta-defensins
Source: BMC Evol Biol. 2008 Apr 16;8:113. doi: 10.1186/1471-2148-8-113 (PMC2373304; doi:10.1186/1471-2148-8-113)
Supplement: Additional file 5 — Supplementary Table 4 DEFB127 haplotypes. [file 1471-2148-8-113-S5.doc]

Supplementary table 3 – DEFB127 haplotypes

Polymorphic sites used for allele-specific PCR are shown in bold, and sites changing an amino acid are shown underlined. Site 15 is a 10bp deletion spanning site 16, occurs in one individual and was ignored in further analyses because of the disruption of site 16.

| Site | **1** | 2 | 3 | 4 | 5 | 6 | 7 | 8 | 9 | 10 | 11 | 12 | 13 | 14 | 15 | 16 | 17 | 18 | 19 | **20** | 21 | 22 | 23 | 24 | 25 | 26 | 27 | 28 | **29** | 30 | 31 | 32 | 33 | 34 | 35 | **36** |
| --- | --- | --- | --- | --- | --- | --- | --- | --- | --- | --- | --- | --- | --- | --- | --- | --- | --- | --- | --- | --- | --- | --- | --- | --- | --- | --- | --- | --- | --- | --- | --- | --- | --- | --- | --- | --- |
| Build 35 chr 20 | **84568** | 84721 | 84936 | 84983 | 85114 | 85128 | 85293 | 85310 | 85374 | 85454 | 85674 | 85855 | 85900 | 85933 | 85940 | 85949 | 86004 | 86125 | 86148 | **86180** | 86221 | 86460 | 86520 | 86527 | 87154 | 87173 | 87362 | 87409 | **87411** | 87419 | 87476 | 87538 | 87576 | 87577 | 87745 | **87841** |
| rs | **610848** | 12185780 | - | - | 13043289 | 1935387 | 6108051 | 6086226 | 11905393 | 12480529 | 2117453 | - | 1434789 | 1434790 | - | - | 6055356 | 2298108 | 2298109 | **3216039** | - | 6077288 | - | 1858594 | 12626088 | 11087789 | 6055460 | 12624951 | **-** | 16995668 | 12624954 | - | 16995685 | - | 4142304 | **4142303** |
| NA02476 (SSA) | A | T | - | T | A | A | C | C | C | T | G | C | T | C | i | C | G | G | C | C | T | A | T | T | A | T | G | G | A | C | G | A | C | G | T | A |
|  | A | T | - | T | A | G | C | C | C | T | G | C | T | G | i | C | C | G | C | C | C | A | T | T | A | T | A | G | A | T | G | A | C | G | C | T |
| NA08688 (SSA) | A | T | - | T | A | G | C | C | T | T | G | C | T | G | i | C | G | G | C | C | C | A | T | T | A | T | A | G | A | C | G | A | C | G | C | T |
|  | A | T | - | T | A | G | C | C | C | T | A | C | T | G | i | C | G | G | C | - | C | A | T | T | A | T | A | G | A | C | G | A | C | G | C | T |
| HAM001  (SSA) | A | T | - | T | A | A | C | C | C | T | G | C | T | C | i | C | C | G | C | C | C | A | T | T | A | T | G | G | A | C | G | A | C | G | T | A |
|  | A | T | - | T | A | G | C | C | C | T | G | C | T | G | i | C | C | G | C | C | C | A | T | T | A | T | A | G | A | C | G | A | C | G | C | T |
| HAM006  (SSA) | A | T | - | T | A | G | C | C | C | T | G | C | T | G | i | C | G | G | C | C | C | A | T | T | A | T | A | G | A | C | G | A | C | G | C | T |
|  | A | T | - | T | A | G | C | C | C | T | G | C | T | G | d | d | G | G | C | C | C | A | T | T | A | T | A | G | A | C | G | A | C | G | C | T |
| HAM008  (SSA) | A | T | - | T | A | G | C | C | C | T | A | C | T | G | i | C | G | G | C | - | C | A | T | T | A | T | A | G | A | C | G | A | C | G | C | T |
|  | A | T | - | T | A | G | C | C | C | T | G | C | T | G | i | C | G | G | C | C | C | A | T | T | A | T | A | G | A | C | G | G | C | G | C | T |
| NA02064 (SSA) | A | T | - | T | A | A | C | C | C | T | G | C | T | C | i | C | G | G | C | C | C | A | T | T | A | T | G | G | A | C | G | A | C | G | T | A |
|  | A | T | - | T | A | A | C | C | C | T | G | C | T | C | i | C | G | G | C | C | C | A | T | T | A | T | G | G | A | C | G | A | C | G | T | A |
| NA00522 (SSA) | A | T | - | T | A | A | C | C | C | T | G | C | T | C | i | C | C | G | C | C | C | A | T | T | G | T | G | G | A | C | G | A | C | G | T | A |
|  | A | T | - | T | A | G | C | C | C | T | A | C | T | G | i | C | G | G | C | - | C | A | T | T | G | T | A | G | A | C | G | A | C | G | C | T |
| IHLAD031  (IA) | A | T | - | T | A | G | C | C | C | T | A | C | T | G | i | C | G | G | C | C | C | A | T | T | A | T | A | G | A | C | G | A | C | G | C | A |
|  | A | T | - | T | A | G | C | C | C | T | A | C | T | G | i | C | G | G | C | C | C | A | T | T | A | T | A | G | A | C | G | A | C | G | C | A |
| BUR-E  (IA) | A | T | - | T | A | G | C | C | C | T | A | C | T | G | i | C | G | G | C | - | C | A | T | T | A | T | A | G | A | C | G | A | C | G | C | T |
|  | A | T | - | T | A | G | C | C | C | T | A | C | T | G | i | C | G | G | C | - | C | A | T | T | A | T | A | G | A | C | G | A | C | G | C | T |
| WOO-EM  (IA) | A | T | - | T | A | G | C | C | C | T | A | C | T | G | i | C | G | G | C | - | C | A | T | T | A | T | A | G | A | C | G | A | C | G | C | T |
|  | A | T | - | T | A | G | C | C | C | T | A | C | T | G | i | C | G | G | C | - | C | A | T | T | A | T | A | G | A | C | G | A | C | G | C | T |
| HAY-BD  (IA) | A | C | A | T | G | A | C | A | C | G | G | C | G | G | i | C | G | T | T | C | C | G | T | C | G | C | G | A | A | C | A | A | A | G | C | A |
|  | A | C | A | T | G | A | C | A | C | G | G | C | G | G | i | C | G | T | T | C | C | G | T | C | G | C | G | A | A | C | A | A | A | G | C | A |
| IHLAD036  (IA) | A | T | - | T | A | G | C | C | C | T | A | C | T | G | i | C | G | G | C | - | C | A | T | T | A | T | A | G | A | C | G | A | C | G | C | T |
|  | A | T | - | T | A | G | C | C | C | T | G | C | T | G | i | C | G | G | C | C | C | A | T | T | A | T | A | G | A | C | G | A | C | G | C | T |
| NON-L  (IA) | A | C | A | T | G | A | C | A | C | G | G | C | G | G | i | C | G | T | T | C | C | G | T | C | G | C | G | A | A | C | A | A | A | G | C | A |
|  | A | T | - | T | A | G | C | C | C | T | A | C | T | G | i | C | G | G | C | - | C | A | T | T | A | T | A | G | A | C | G | A | C | G | C | T |
| WON-M  (IA) | A | T | - | T | A | G | C | C | C | T | A | C | T | G | i | C | G | G | C | - | C | A | T | T | A | T | A | G | A | C | G | A | C | G | C | T |
|  | A | T | - | T | A | G | C | C | C | T | A | C | T | G | i | C | G | G | C | - | C | A | T | T | A | T | A | G | A | C | G | A | C | G | C | T |
| WON-I  (IA) | A | T | - | T | A | G | C | C | C | T | G | C | T | G | i | C | G | G | C | - | C | A | T | T | A | T | A | G | A | C | G | A | C | G | C | T |
|  | A | T | - | T | A | G | C | C | C | T | G | C | T | G | i | C | G | G | C | - | C | A | T | T | A | T | A | G | A | C | G | A | C | G | C | T |
| WON-C  (IA) | C | T | - | T | A | A | T | C | C | T | G | C | T | G | i | C | G | G | C | C | C | A | T | T | A | T | G | G | A | C | G | A | C | G | C | A |
|  | A | T | - | T | A | G | C | C | C | T | A | C | T | G | i | C | G | G | C | - | C | A | T | T | A | T | A | G | A | C | G | A | C | G | C | T |
| HAY-KJ  (IA) | A | C | A | T | G | A | C | A | C | G | G | C | G | G | i | C | G | T | T | C | C | G | T | C | G | C | G | A | A | C | A | A | A | G | C | A |
|  | A | T | - | T | A | G | C | C | C | T | A | C | T | G | i | C | G | G | C | - | C | A | T | T | A | T | A | G | A | C | G | A | C | G | C | T |
| NA10469 (MB) | A | T | - | T | A | G | C | C | C | T | A | C | T | G | i | C | G | G | C | - | C | A | T | T | A | T | A | G | A | C | G | A | C | G | C | T |
|  | A | T | - | T | A | G | C | C | C | T | G | C | T | G | i | T | G | G | C | C | C | A | T | T | A | T | A | G | A | C | G | A | C | G | C | T |
| NA10470 (MB) | A | T | - | T | A | G | C | C | C | T | G | C | T | G | i | T | G | G | C | C | C | A | T | T | A | T | A | G | A | C | G | A | C | A | C | T |
|  | A | T | - | T | A | G | C | C | C | T | A | C | T | G | i | C | G | G | C | - | C | A | T | T | A | T | A | G | A | C | G | A | C | G | C | T |
| NA10471 (MB) | A | T | - | T | A | G | C | C | C | T | G | C | T | G | i | C | G | G | C | C | C | A | T | T | A | T | A | G | A | C | G | A | C | A | C | T |
|  | A | T | - | T | A | G | C | C | C | T | A | C | T | G | i | C | G | G | C | - | C | A | T | T | A | T | A | G | A | C | G | A | C | G | C | T |
| NA10472 (MB) | A | T | - | T | A | G | C | C | C | T | G | C | T | G | i | C | G | G | C | - | C | A | T | T | A | T | A | G | A | **C** | G | A | C | G | C | T |
|  | A | T | - | T | A | G | C | C | C | T | G | C | T | G | i | C | G | G | C | C | C | A | T | T | A | T | A | G | A | **T** | G | A | C | G | C | T |
| NA10473 (MB) | A | T | - | T | A | G | C | C | C | T | A | C | T | G | i | C | G | G | C | - | C | A | T | T | A | T | A | G | A | C | G | A | C | G | C | T |
|  | A | T | - | T | A | G | C | C | C | T | G | C | T | G | i | C | G | G | C | C | C | A | T | T | A | T | A | G | A | C | G | A | C | G | C | T |
| NA10492 (MB) | A | T | - | T | A | G | C | C | C | T | G | C | T | G | i | C | G | G | C | C | C | A | C | T | A | T | G | G | T | C | G | A | C | G | C | T |
|  | A | T | - | T | A | G | C | C | C | T | G | C | T | G | i | T | G | G | C | C | C | A | T | T | A | T | G | G | A | C | G | A | C | G | C | T |
| NA10493 (MB) | A | T | - | T | A | G | C | C | C | T | G | C | T | G | i | T | C | G | C | C | C | A | T | T | A | T | A | G | A | C | G | A | C | G | C | T |
|  | A | T | - | T | A | G | C | C | C | T | G | C | T | C | i | C | G | G | C | C | C | A | T | T | A | T | G | G | A | C | G | A | C | G | T | A |
| NA10494 (MB) | A | T | - | T | A | G | C | C | C | T | G | C | T | G | i | T | G | G | C | C | C | A | T | T | A | T | A | G | A | C | G | A | C | G | C | T |
|  | A | T | - | T | A | G | C | C | C | T | G | C | T | G | i | C | G | G | C | C | C | A | T | T | A | T | A | G | A | T | G | A | C | G | C | T |
| NA10495 (MB) | C | T | - | T | A | A | T | C | C | T | G | C | T | G | i | C | G | G | C | C | C | A | T | T | A | T | G | G | A | C | G | A | C | G | C | T |
|  | A | C | A | T | G | A | C | A | C | G | G | C | G | G | i | C | G | T | T | C | C | G | T | **C** | G | T | G | A | A | C | A | A | A | G | C | A |
| NA10496 (MB) | A | T | - | T | A | G | C | C | C | T | G | C | T | G | i | T | G | G | C | C | C | A | T | T | A | T | A | G | A | C | G | A | C | G | C | T |
|  | A | T | - | T | A | G | C | C | C | T | G | C | T | G | i | C | G | G | C | C | C | A | T | T | A | T | A | G | T | C | G | A | C | G | C | T |
| CO156  (UK) | A | T | - | T | A | G | C | C | C | T | A | C | T | G | i | C | G | G | C | - | C | A | T | T | A | T | A | G | A | C | G | A | C | G | C | T |
|  | A | C | A | T | G | A | C | A | C | G | G | C | G | G | i | C | G | T | T | C | C | G | T | C | G | C | G | A | A | C | A | A | A | G | C | A |
| CO896  (UK) | A | T | - | T | A | G | C | C | C | T | A | C | T | G | i | C | G | G | C | - | C | A | T | T | A | T | A | G | A | C | G | A | C | G | C | T |
|  | A | C | A | T | G | A | C | A | C | G | G | C | G | G | i | C | G | T | T | C | C | G | T | C | G | C | G | A | A | C | A | A | A | G | C | A |
| CO157  (UK) | A | T | - | T | A | G | C | C | C | T | A | C | T | G | i | C | G | G | C | C | C | A | T | T | A | T | A | G | A | C | G | A | C | G | C | T |
|  | A | C | A | T | G | A | C | A | C | G | G | C | G | G | i | C | G | T | T | C | C | G | T | C | G | C | G | A | A | C | A | A | A | G | **T** | A |
| CO038  (UK) | A | T | - | T | A | G | C | C | C | T | A | C | T | G | i | C | G | G | C | C | C | A | T | T | A | T | A | G | A | C | G | A | C | G | C | T |
|  | A | C | A | T | G | A | C | A | C | G | G | C | G | G | i | C | G | G | C | C | C | A | T | C | G | C | G | A | A | C | A | A | A | G | C | A |
| CO152  (UK) | A | T | - | T | A | G | C | C | C | T | A | C | T | G | i | C | G | G | C | - | C | A | T | T | A | T | A | G | A | C | G | A | C | G | C | T |
|  | A | T | - | T | A | G | C | C | C | T | A | C | T | G | i | C | G | G | C | - | C | A | T | T | A | T | A | G | A | C | G | A | C | G | C | T |
| CO183  (UK) | A | C | A | T | G | A | C | A | C | G | G | C | G | G | i | C | G | T | T | C | C | G | T | C | G | C | G | A | A | C | A | A | A | G | C | A |
|  | A | C | A | T | G | A | C | A | C | G | G | C | G | G | i | C | G | T | T | C | C | G | T | C | G | C | G | A | A | C | A | A | A | G | C | A |
| CO917  (UK) | A | C | A | T | G | A | C | A | C | G | G | C | G | G | i | C | G | T | T | C | C | G | T | C | G | C | G | A | A | C | A | A | A | G | C | A |
|  | A | C | A | T | G | A | C | A | C | G | G | C | G | G | i | C | G | T | T | C | C | G | T | C | G | C | G | A | A | C | A | A | A | G | C | A |
| CO187  (UK) | A | T | - | T | A | G | C | C | C | T | G | T | T | G | i | C | G | G | C | C | C | A | T | T | A | T | A | G | A | C | G | A | C | G | C | T |
|  | A | T | - | T | A | G | C | C | C | T | A | C | T | G | i | C | G | G | C | - | C | A | T | T | A | T | A | G | A | C | G | A | C | G | C | T |
| CO208  (UK) | A | T | - | T | A | G | C | C | C | T | G | C | T | G | i | C | G | G | C | C | C | A | T | T | A | T | A | G | A | C | G | A | C | G | C | T |
|  | A | T | - | T | A | G | C | C | C | T | G | C | T | G | i | C | G | G | C | C | C | A | T | T | A | T | A | G | A | C | G | A | C | G | C | T |
| CO744  (UK) | A | T | - | C | A | G | C | C | C | T | A | C | T | C | i | C | G | G | C | - | C | A | T | T | A | T | A | G | A | C | G | A | C | G | C | T |
|  | A | T | - | T | A | G | C | C | C | T | A | C | T | C | i | C | G | G | C | - | C | A | T | T | A | T | A | G | A | C | G | A | C | G | C | T |
